# Supplementary figures and images for: The role of autophagy in high-fat diet-induced insulin resistance of adipose tissues in mice
Source: PeerJ. 2022 Aug 15;10:e13867. doi: 10.7717/peerj.13867 (PMC9387522; doi:10.7717/peerj.13867)

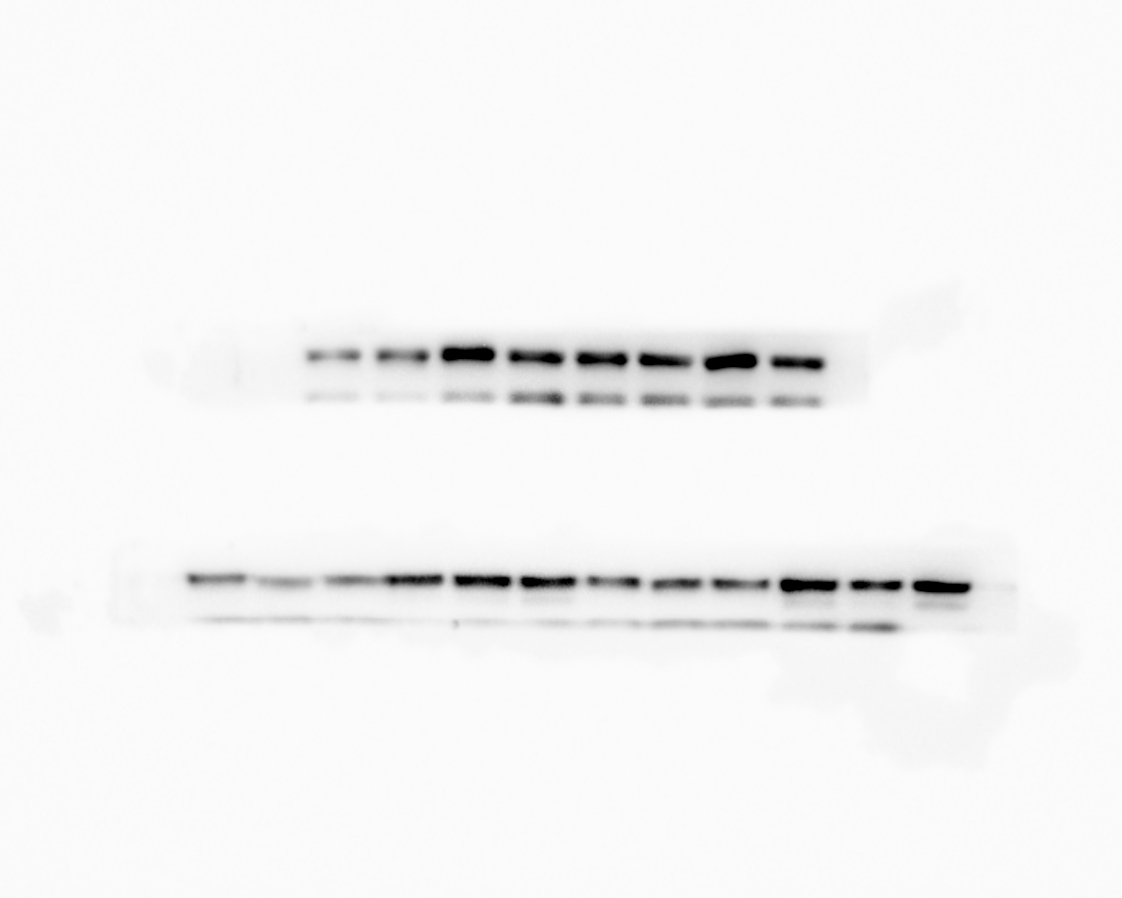

Supplement: Supplemental Information 1 [file peerj-10-13867-s001.zip › uncropped blots in JPEG/figure 1/figure 1A/B6 mice_HFD_8w_AT_Atg5_upper_2018-03-20.jpg]

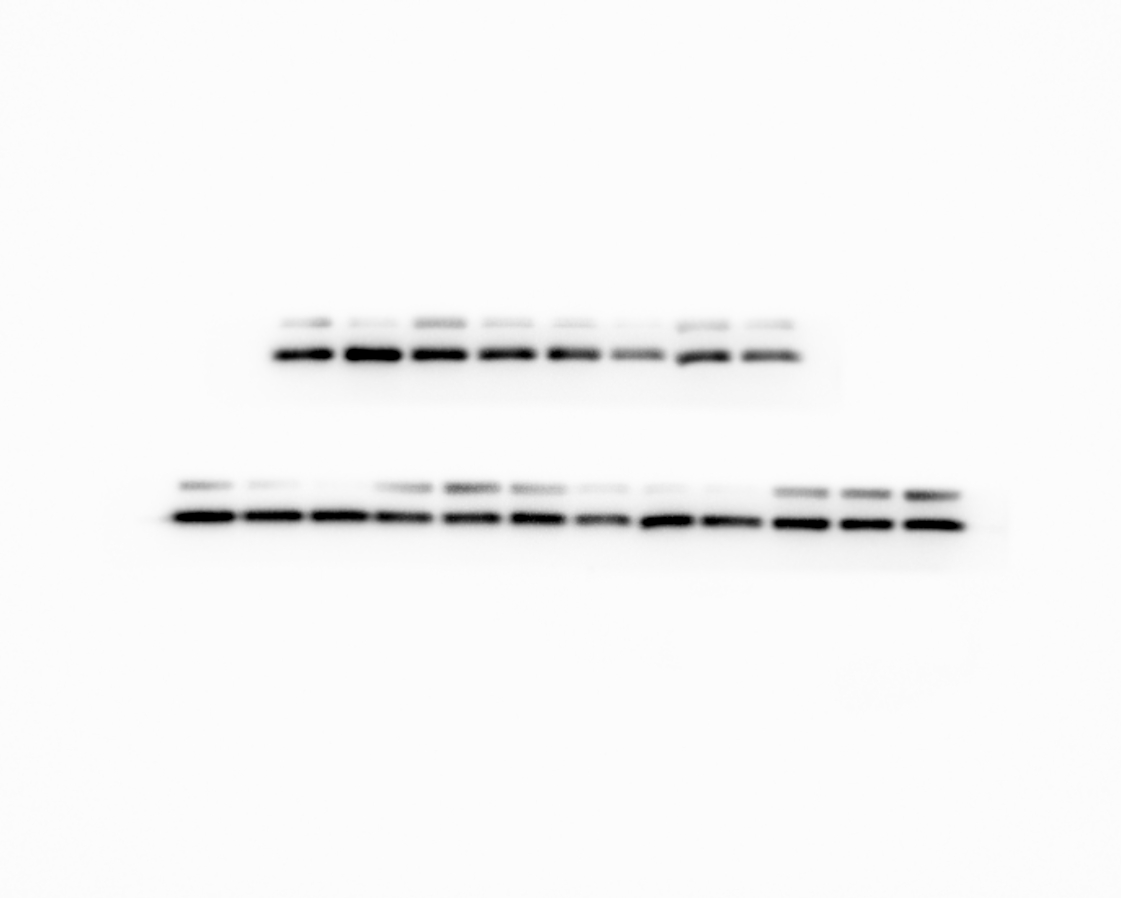

Supplement: Supplemental Information 1 [file peerj-10-13867-s001.zip › uncropped blots in JPEG/figure 1/figure 1A/B6 mice_HFD_8w_AT_GAPDH for LC3 _ Atg5_upper_2018-03-20.jpg]

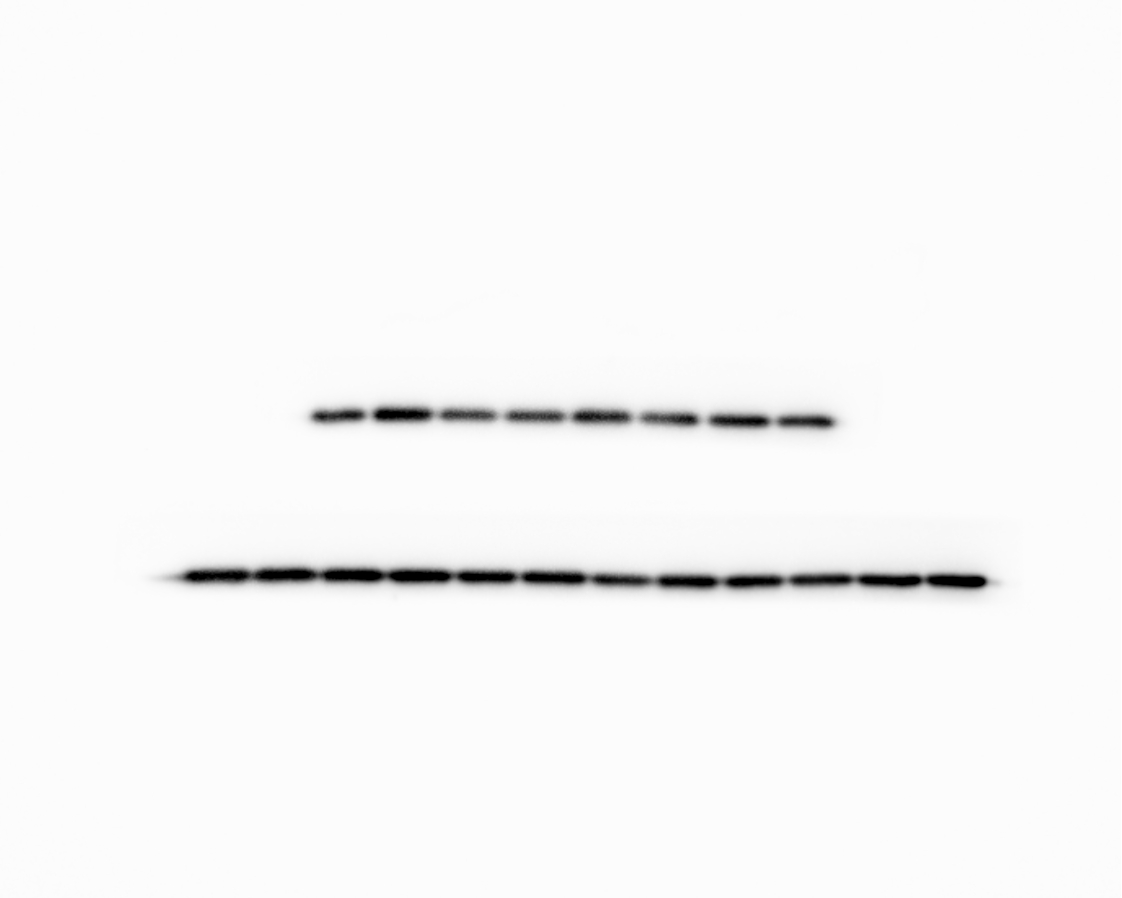

Supplement: Supplemental Information 1 [file peerj-10-13867-s001.zip › uncropped blots in JPEG/figure 1/figure 1A/B6 mice_HFD_8w_AT_GAPDH for p62 _ Rubicon_upper_2018-02-28.jpg]

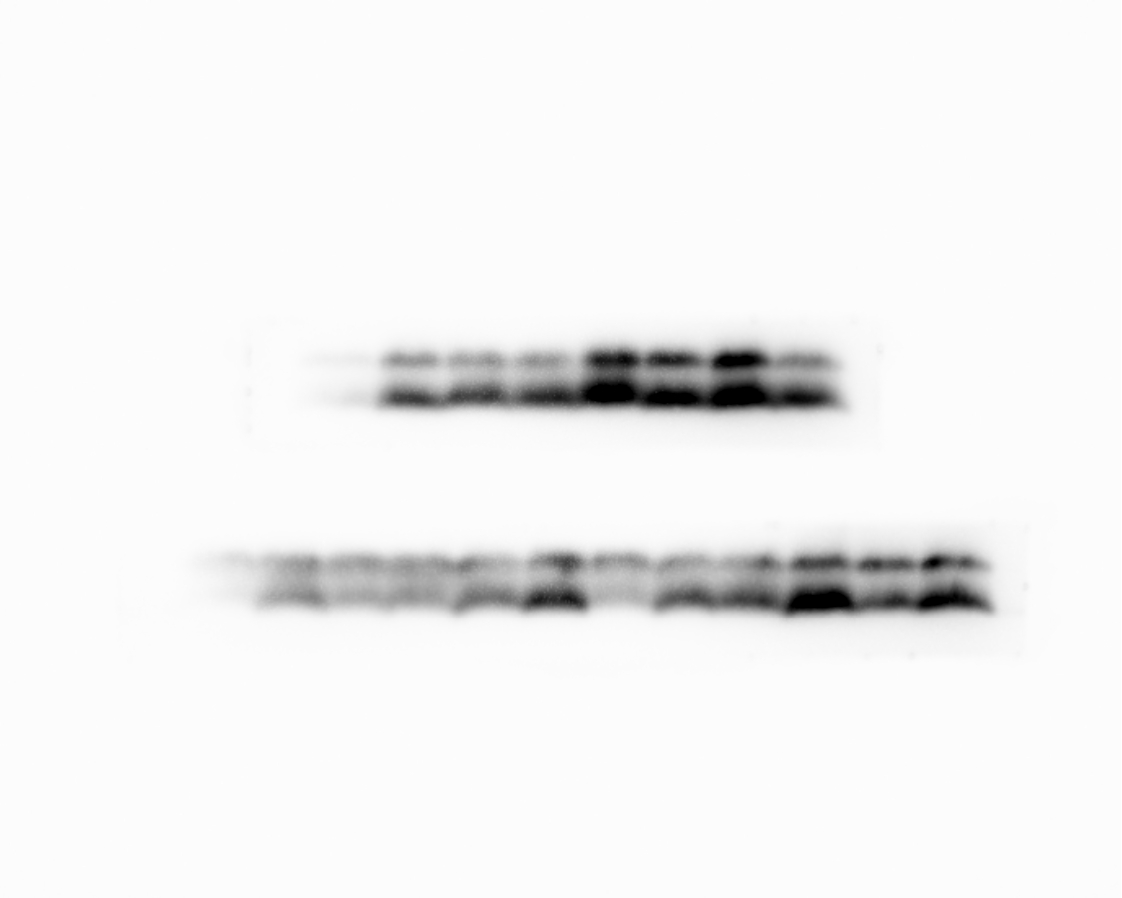

Supplement: Supplemental Information 1 [file peerj-10-13867-s001.zip › uncropped blots in JPEG/figure 1/figure 1A/B6 mice_HFD_8w_AT_LC3_upper_2018-03-20.jpg]

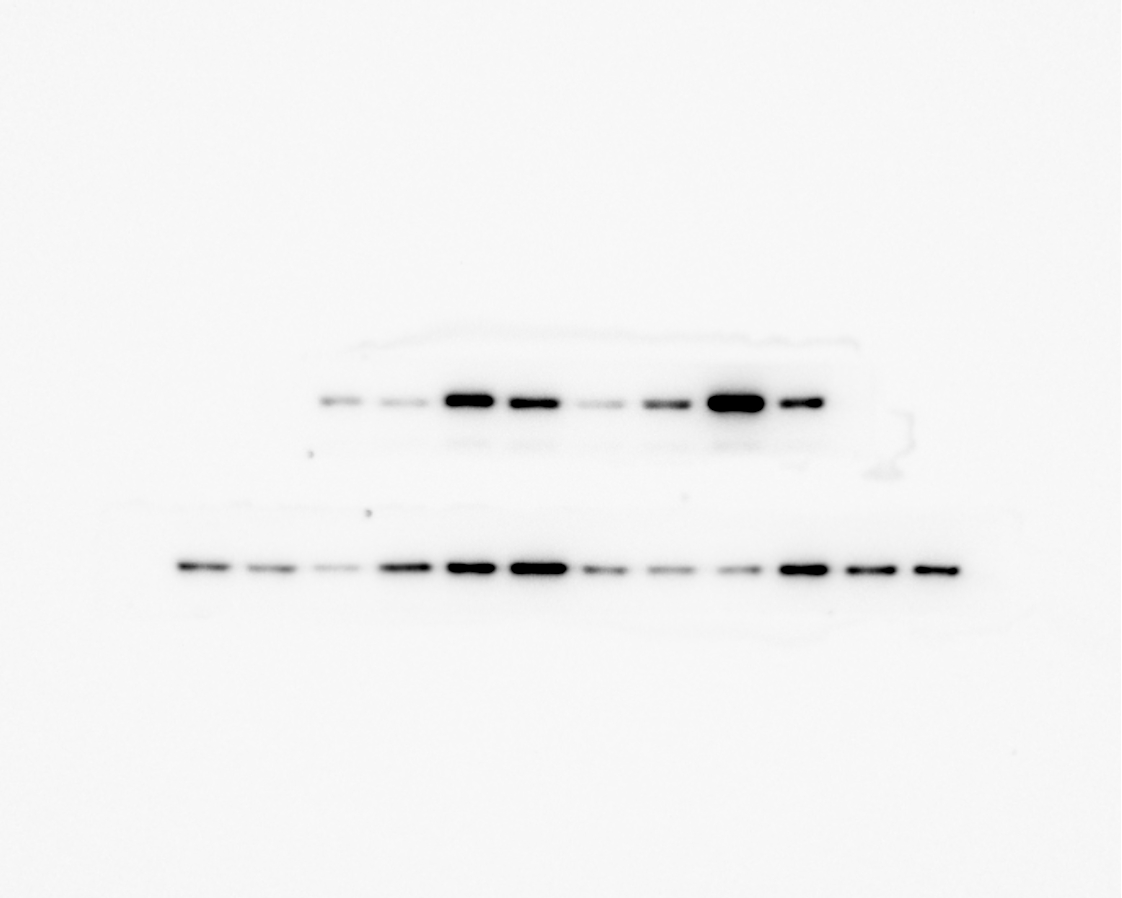

Supplement: Supplemental Information 1 [file peerj-10-13867-s001.zip › uncropped blots in JPEG/figure 1/figure 1A/B6 mice_HFD_8w_AT_p62_upper_2018-02-28.jpg]

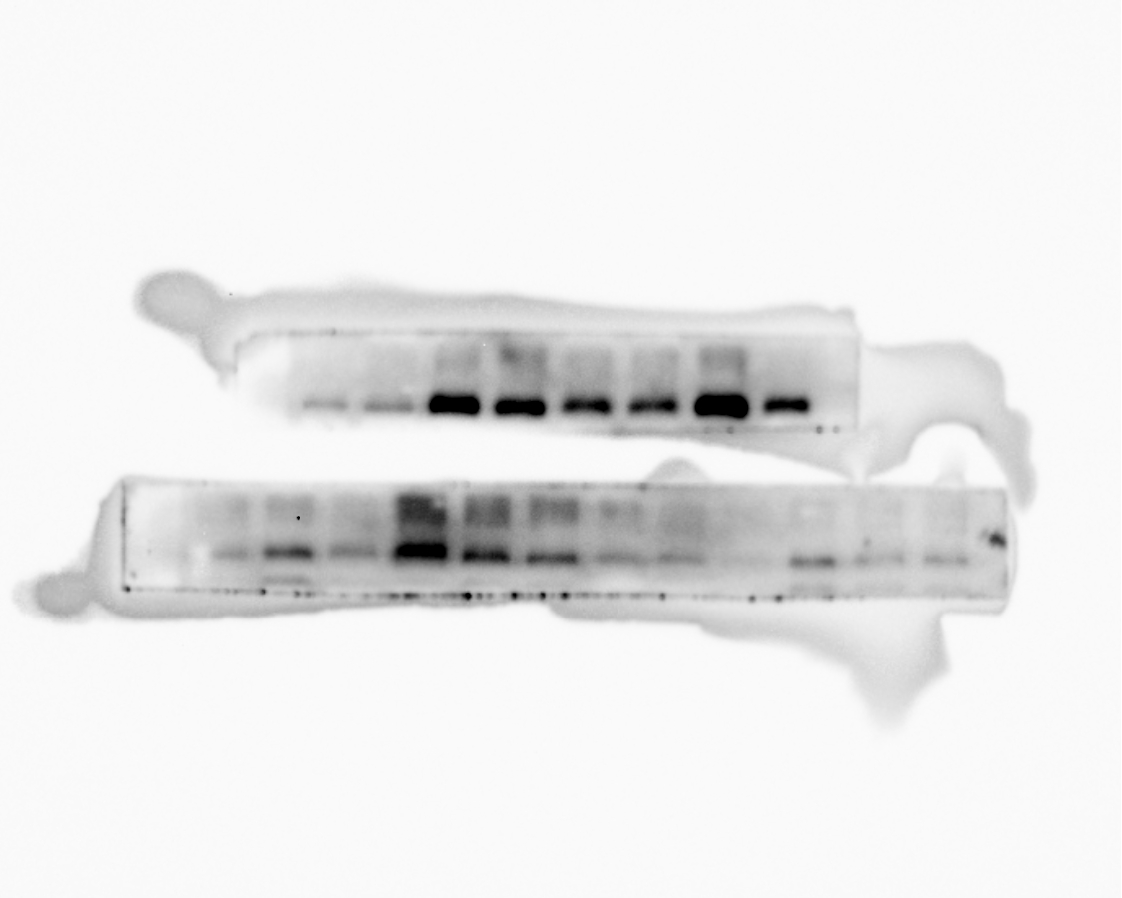

Supplement: Supplemental Information 1 [file peerj-10-13867-s001.zip › uncropped blots in JPEG/figure 1/figure 1A/B6 mice_HFD_8w_AT_Rubicon_upper_2018-02-28.jpg]

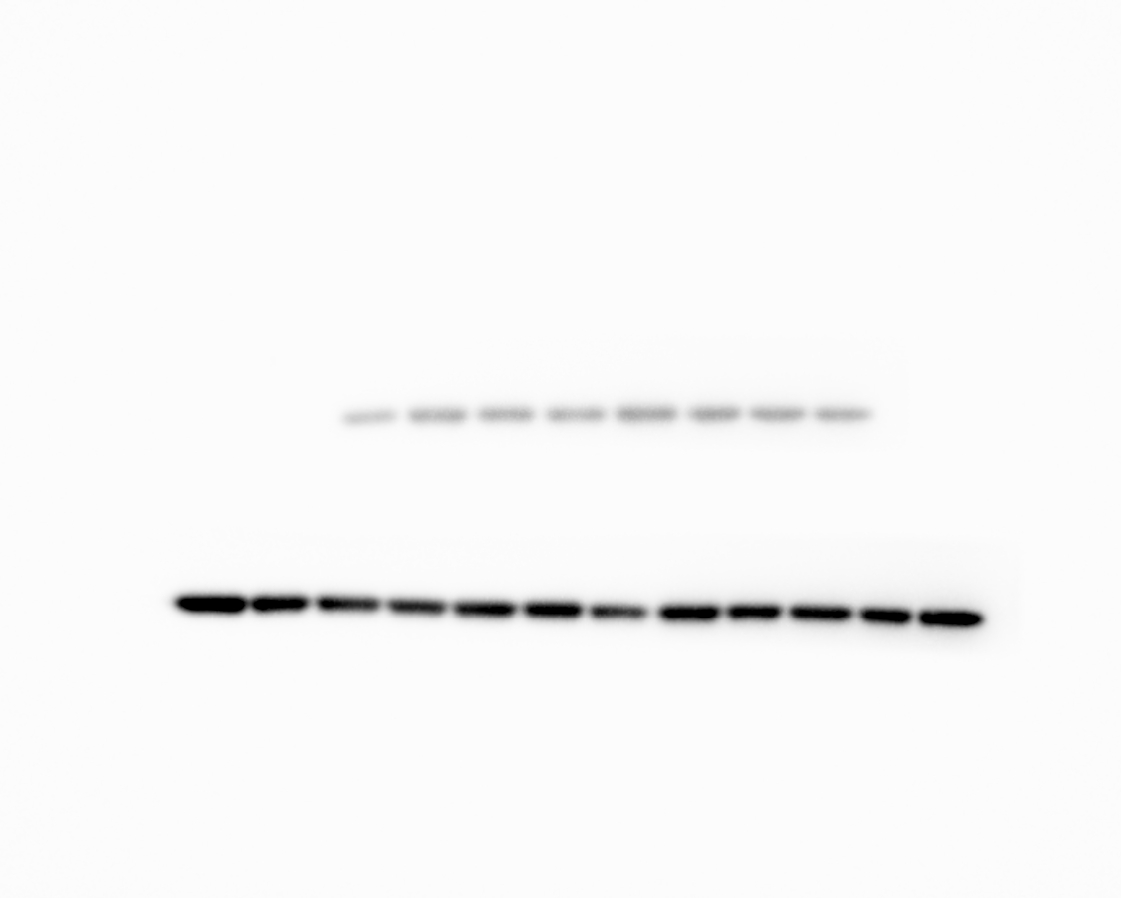

Supplement: Supplemental Information 1 [file peerj-10-13867-s001.zip › uncropped blots in JPEG/figure 1/figure 1C/B6 mice_HFD_16w_AT_GAPDH for Rubicon_left_2018-03-20.jpg]

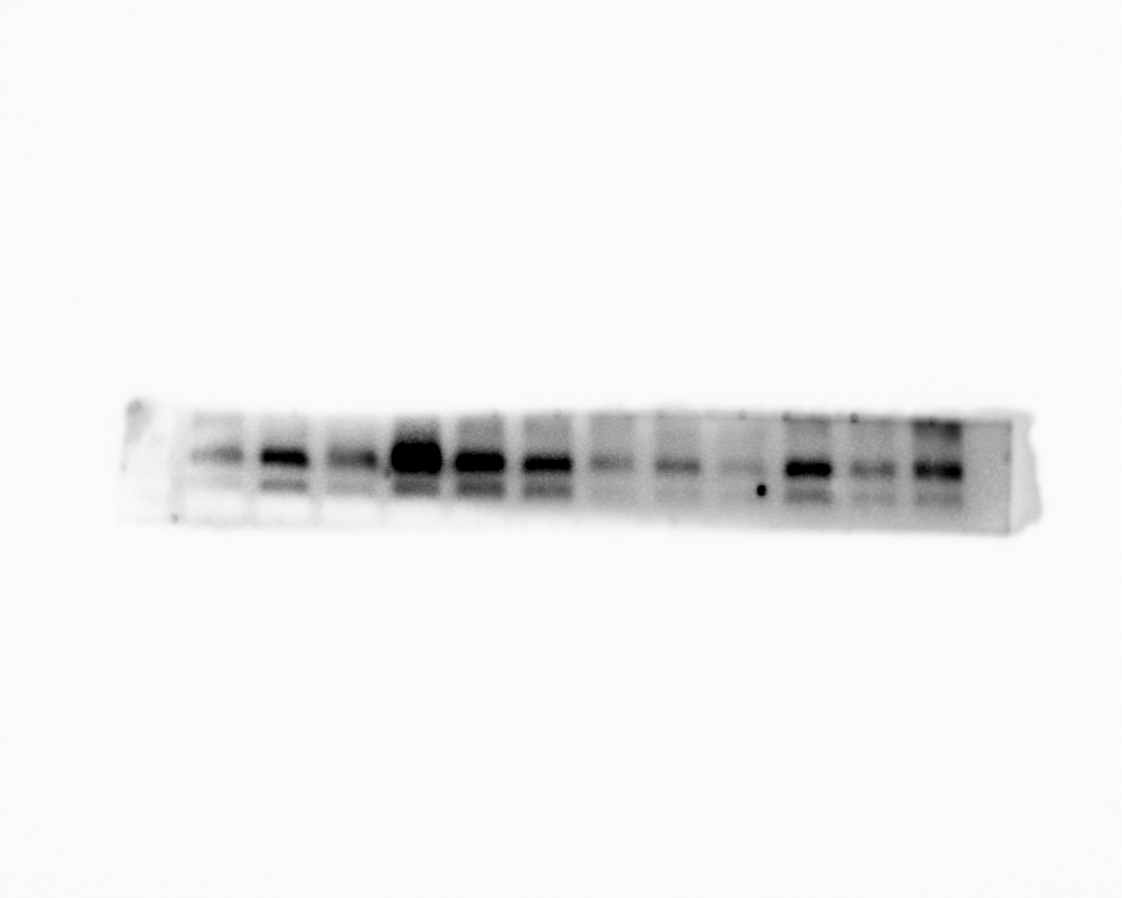

Supplement: Supplemental Information 1 [file peerj-10-13867-s001.zip › uncropped blots in JPEG/figure 1/figure 1C/B6 mice_HFD_16w_AT_Rubicon_2018-03-20.jpg]

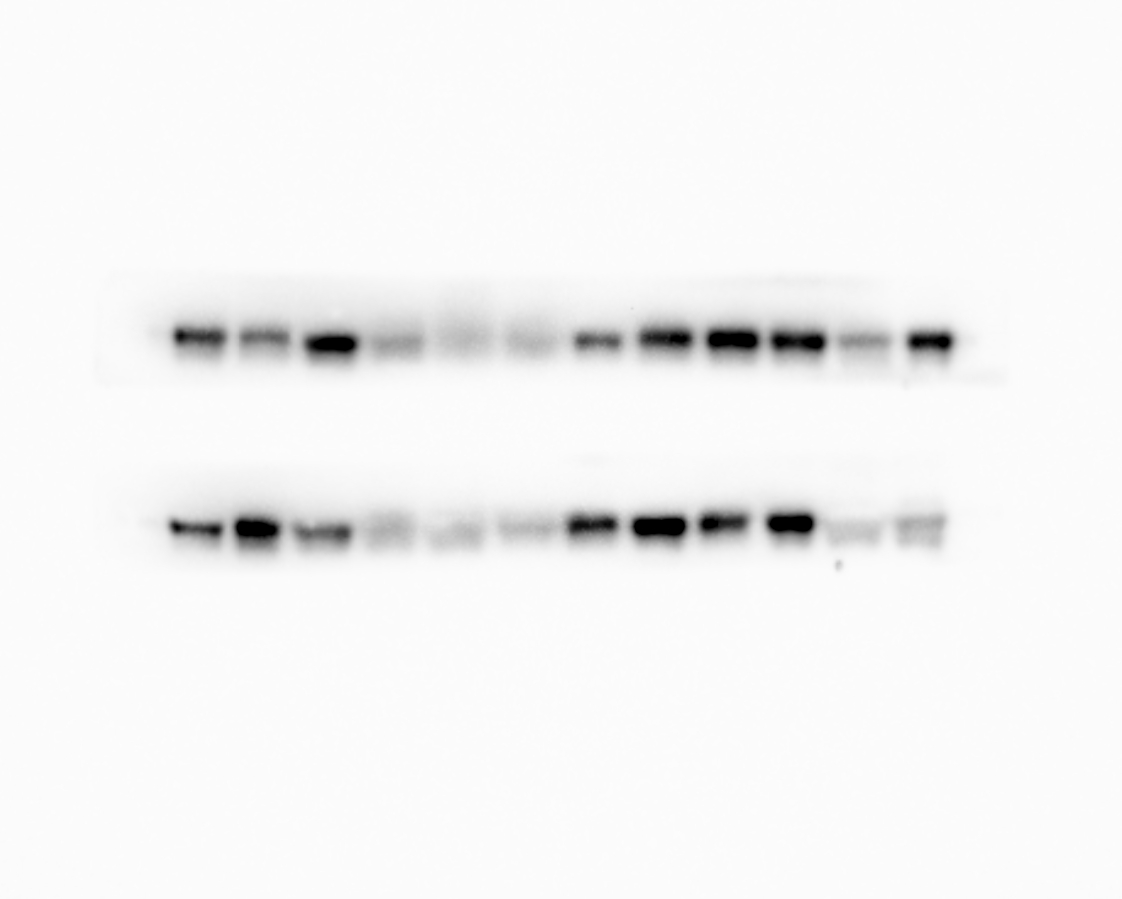

Supplement: Supplemental Information 1 [file peerj-10-13867-s001.zip › uncropped blots in JPEG/figure 1/figure 1F/B6 mice_HFD_16w_AT_+-Ins_Akt_2018-02-03.jpg]

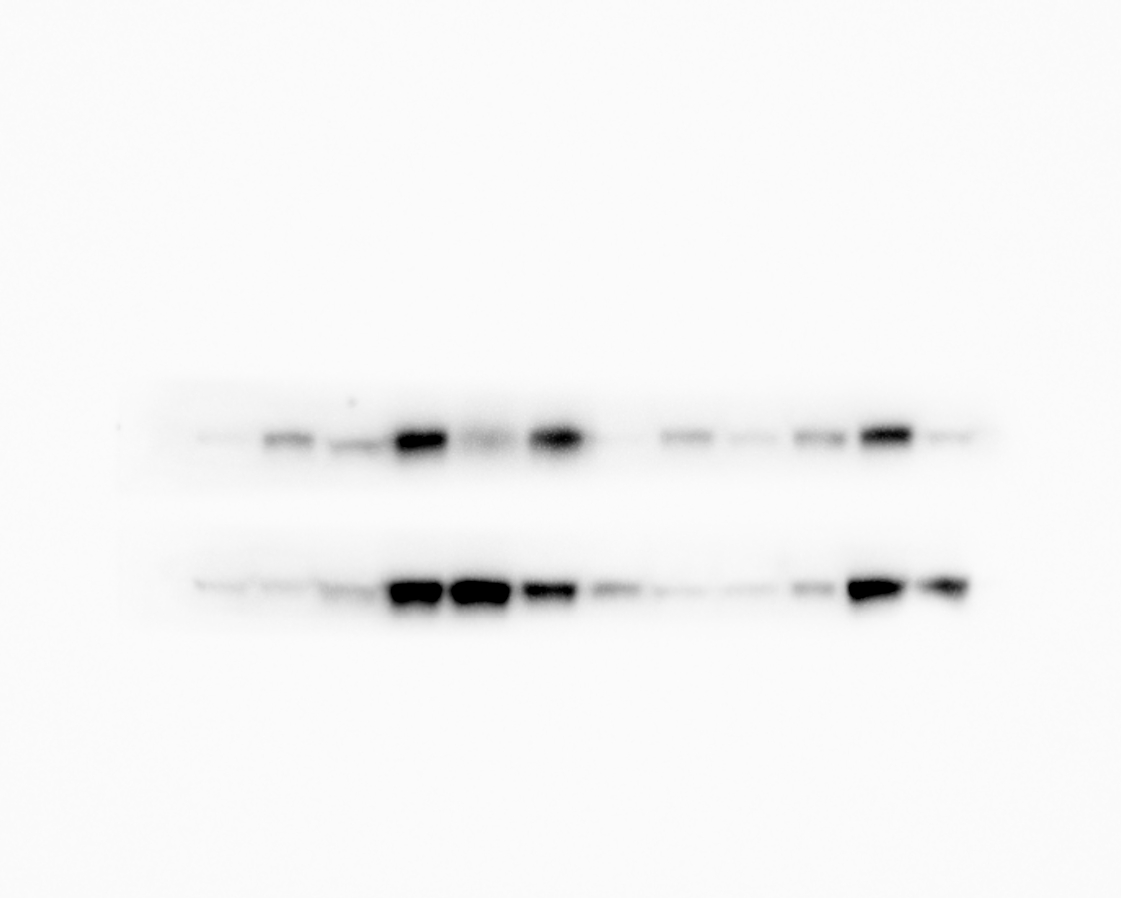

Supplement: Supplemental Information 1 [file peerj-10-13867-s001.zip › uncropped blots in JPEG/figure 1/figure 1F/B6 mice_HFD_16w_AT_+-Ins_p-Akt_2018-02-02.jpg]

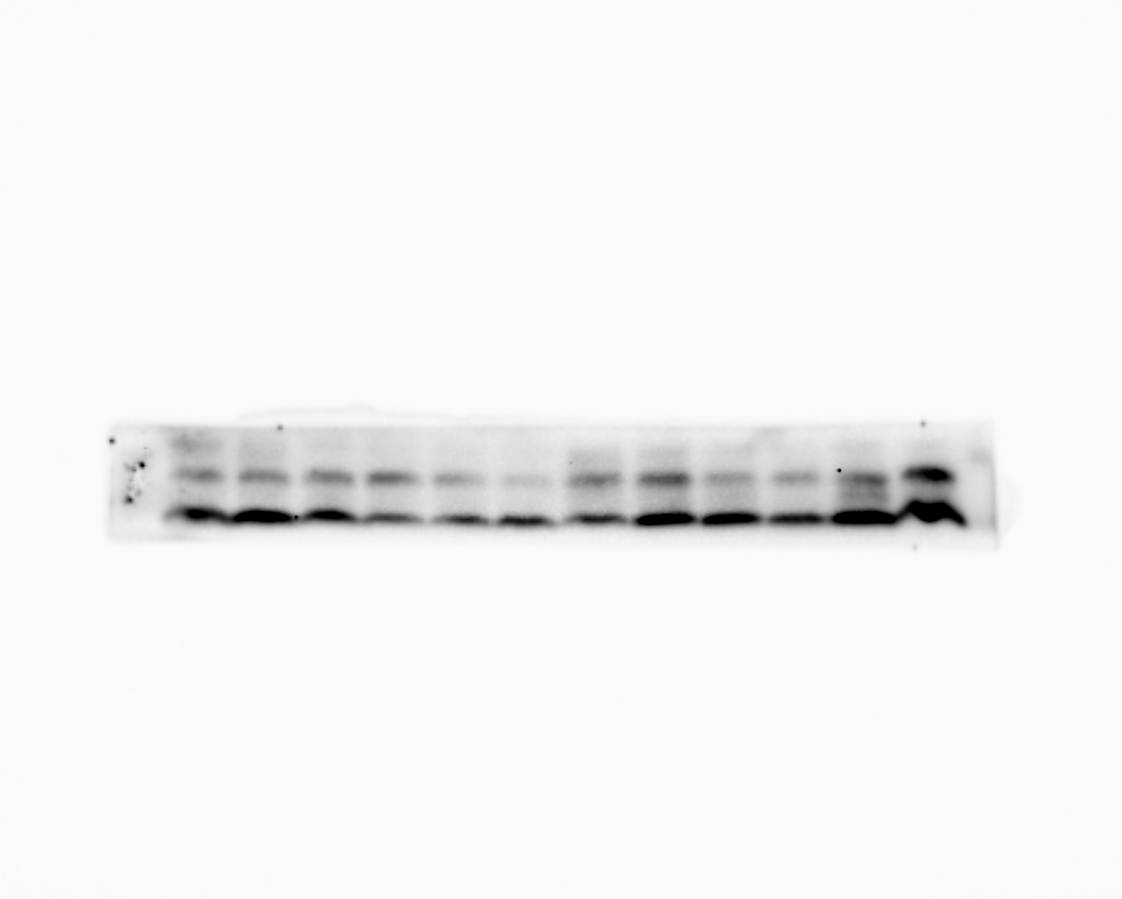

Supplement: Supplemental Information 1 [file peerj-10-13867-s001.zip › uncropped blots in JPEG/figure 1/figure 1H/B6 mice_HFD_16w_AT_CC3_2019-06-02.jpg]

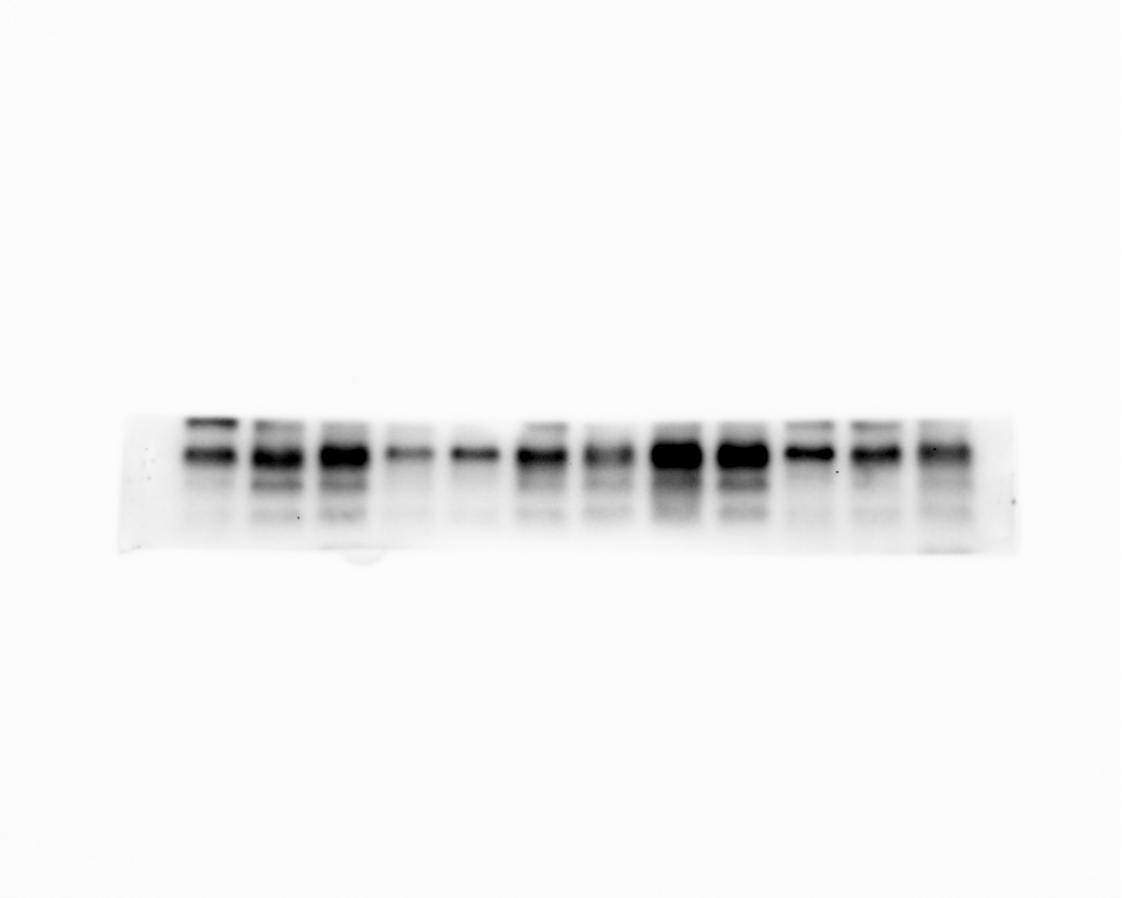

Supplement: Supplemental Information 1 [file peerj-10-13867-s001.zip › uncropped blots in JPEG/figure 1/figure 1H/B6 mice_HFD_16w_AT_CHOP_2019-05-27.jpg]

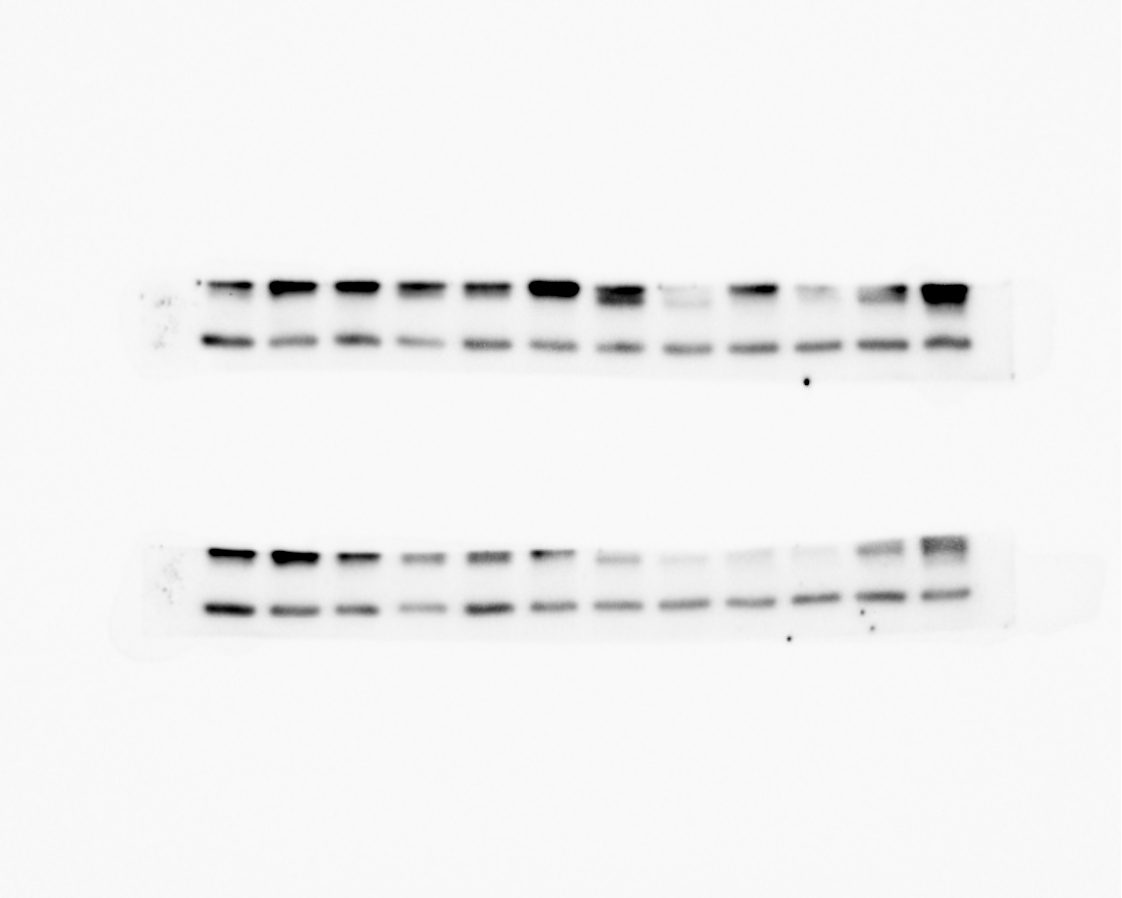

Supplement: Supplemental Information 1 [file peerj-10-13867-s001.zip › uncropped blots in JPEG/figure 1/figure 1H/B6 mice_HFD_16w_AT_GAPDH for CC3_upper_2019-06-02.jpg]

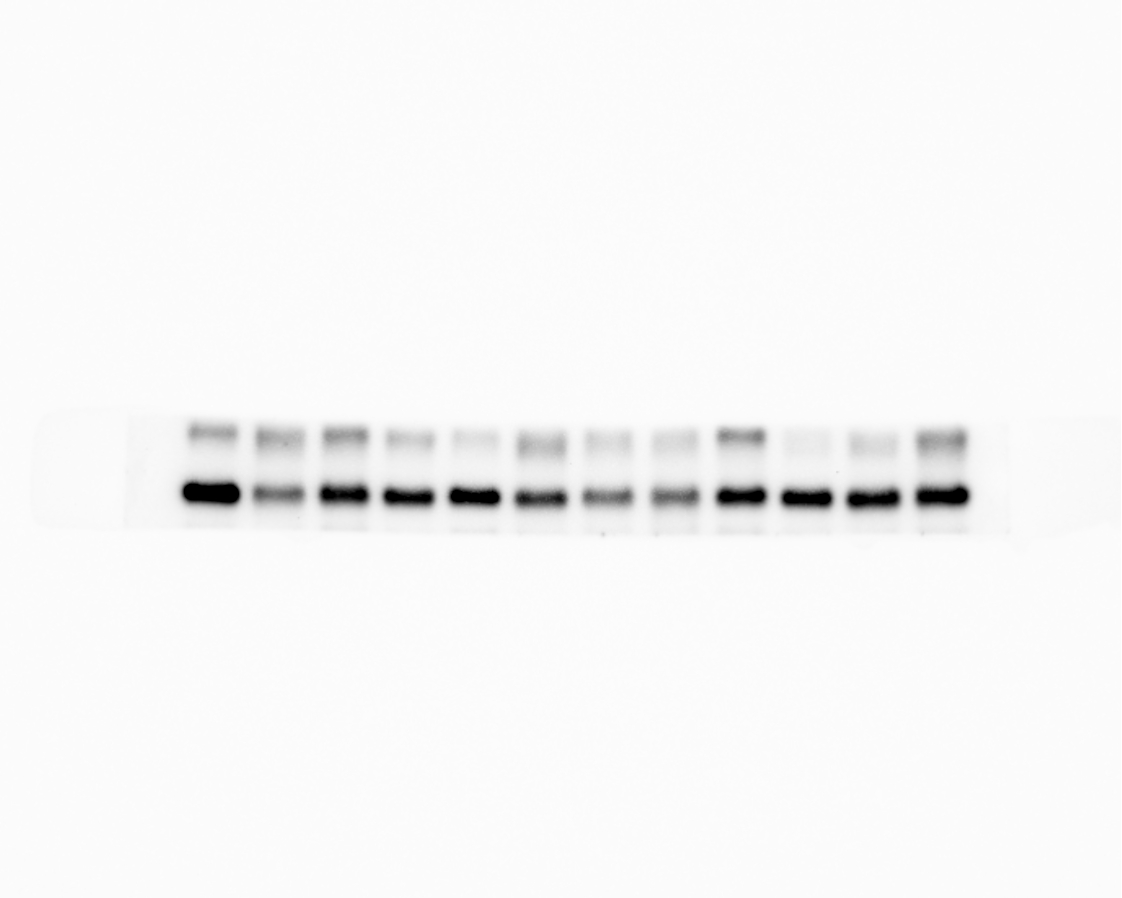

Supplement: Supplemental Information 1 [file peerj-10-13867-s001.zip › uncropped blots in JPEG/figure 1/figure 1H/B6 mice_HFD_16w_AT_GAPDH for CHOP_2019-05-27.jpg]

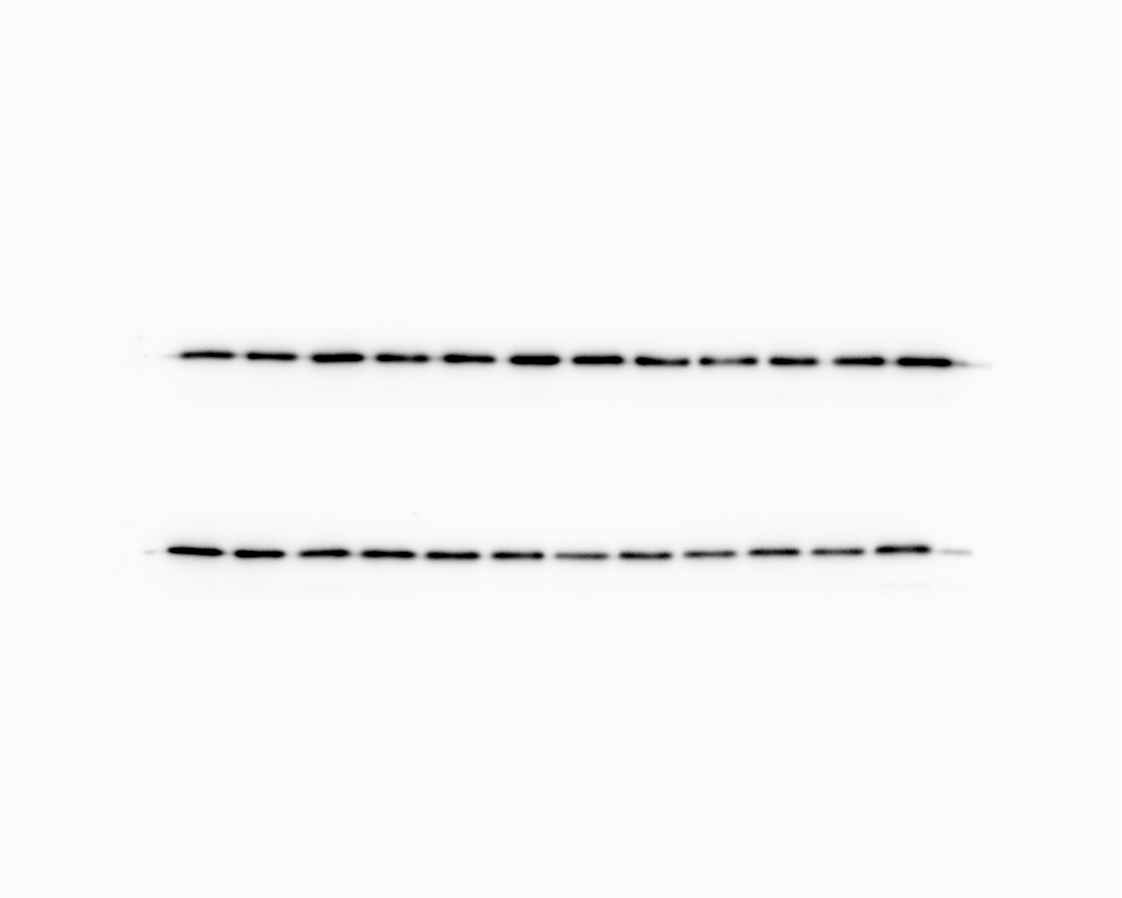

Supplement: Supplemental Information 1 [file peerj-10-13867-s001.zip › uncropped blots in JPEG/figure 2/figure 2A/3T3-L1_1.2FFA_+-baf_GAPDH for LC3_upper_2019-05-08.jpg]

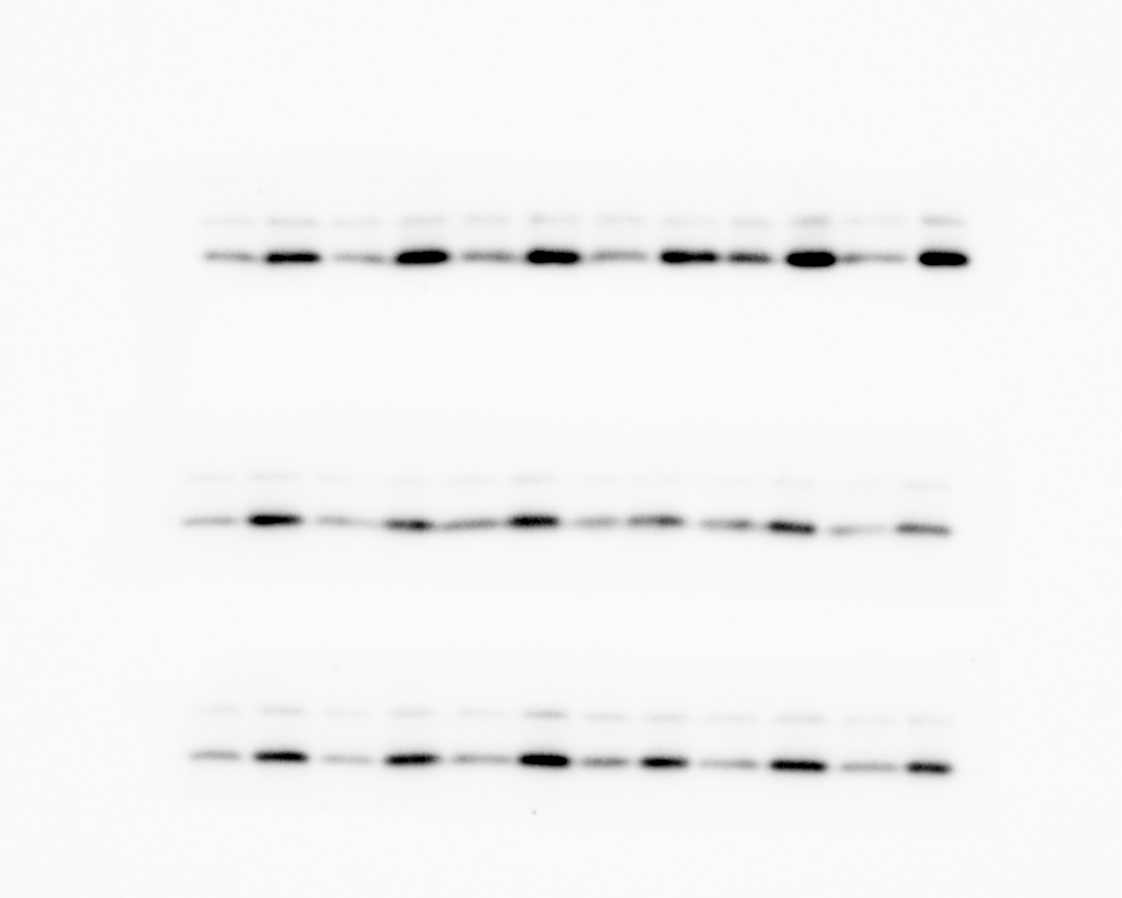

Supplement: Supplemental Information 1 [file peerj-10-13867-s001.zip › uncropped blots in JPEG/figure 2/figure 2A/3T3-L1_1.2FFA_+-baf_LC3_lower_2019-05-08.jpg]

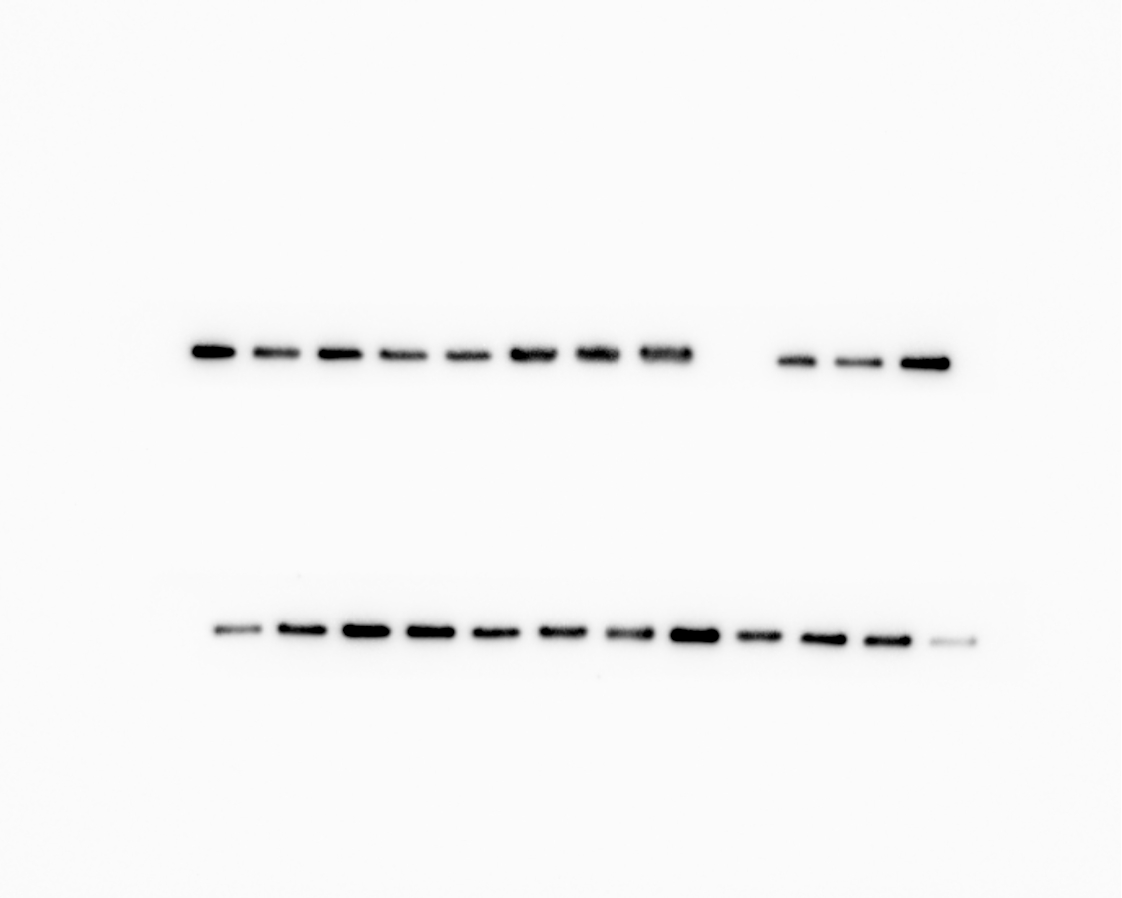

Supplement: Supplemental Information 1 [file peerj-10-13867-s001.zip › uncropped blots in JPEG/figure 3/figure 3A/B6 mice_CQ_24hr_AT_GAPDH for LC3_upper_2019-04-18.jpg]

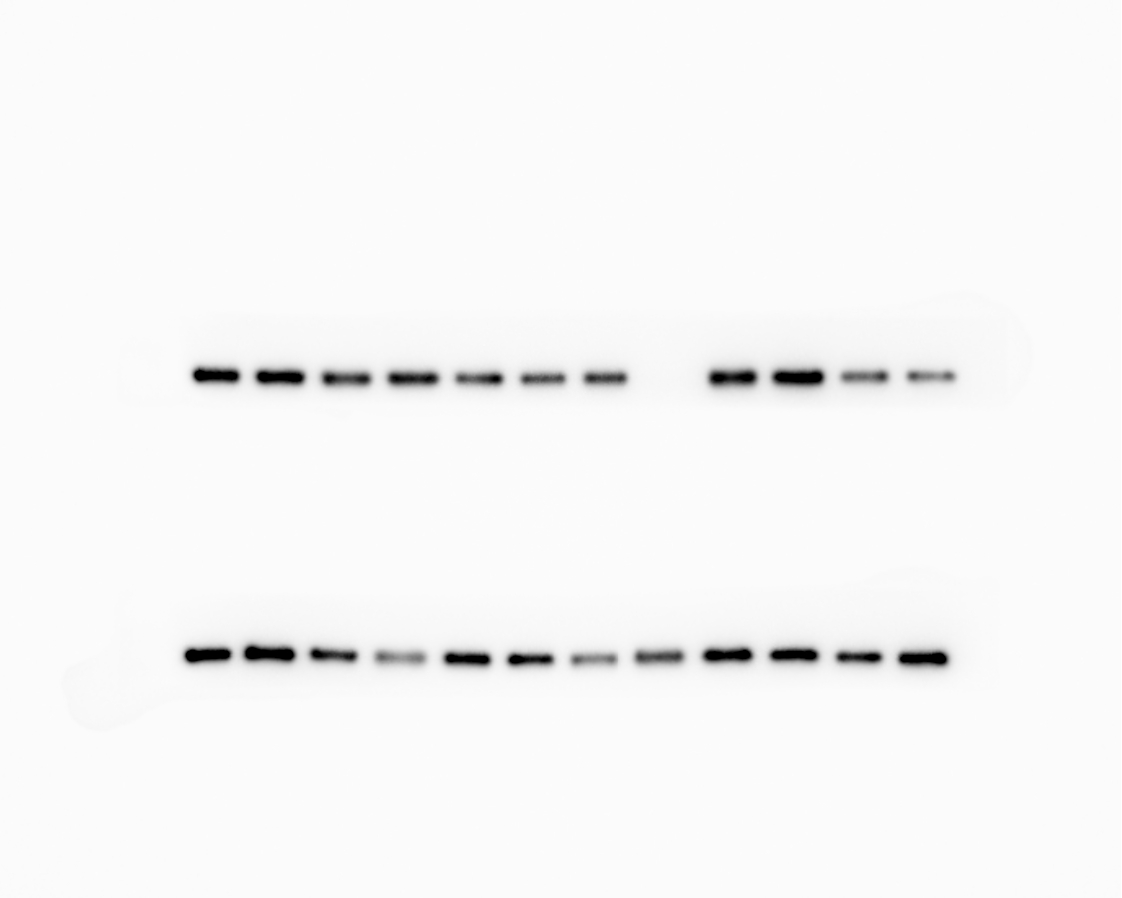

Supplement: Supplemental Information 1 [file peerj-10-13867-s001.zip › uncropped blots in JPEG/figure 3/figure 3A/B6 mice_CQ_24hr_AT_GAPDH for p62_upper_2019-04-16.jpg]

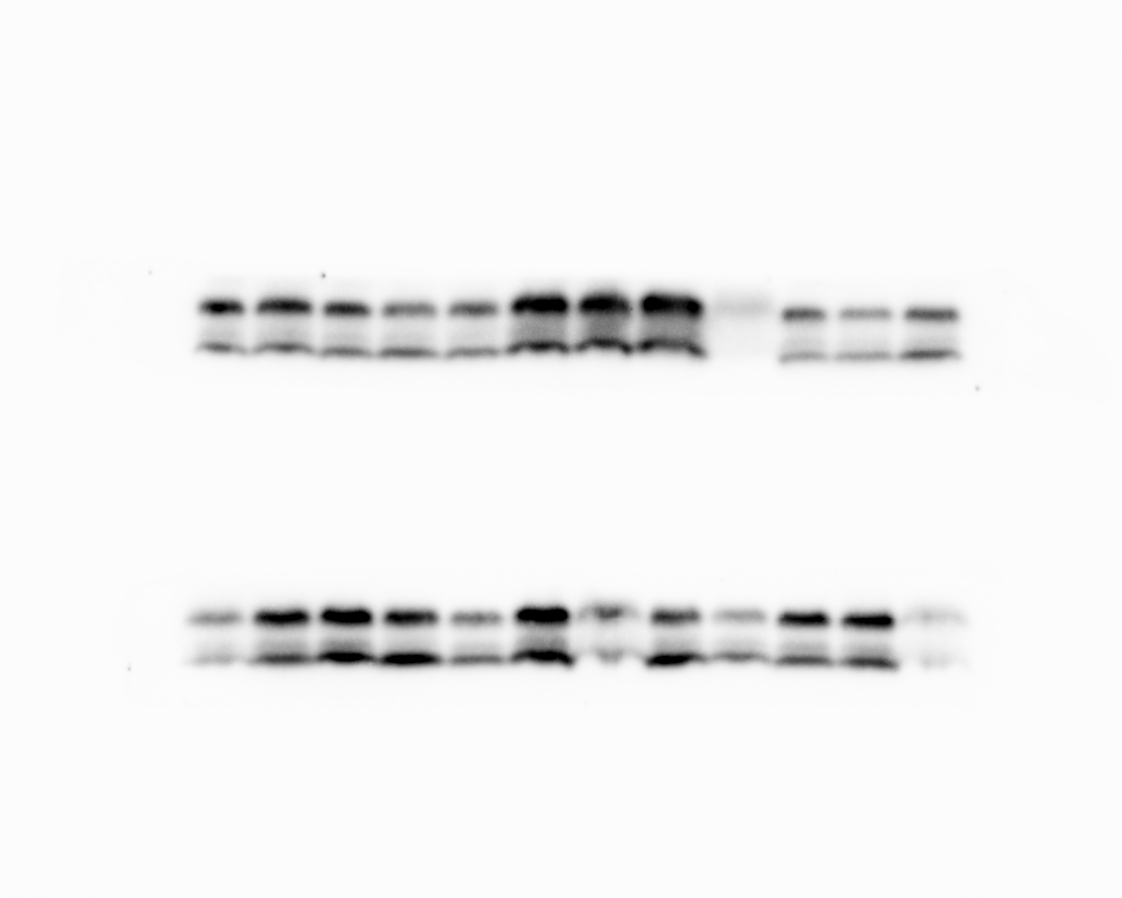

Supplement: Supplemental Information 1 [file peerj-10-13867-s001.zip › uncropped blots in JPEG/figure 3/figure 3A/B6 mice_CQ_24hr_AT_LC3_upper_2019-04-16.jpg]

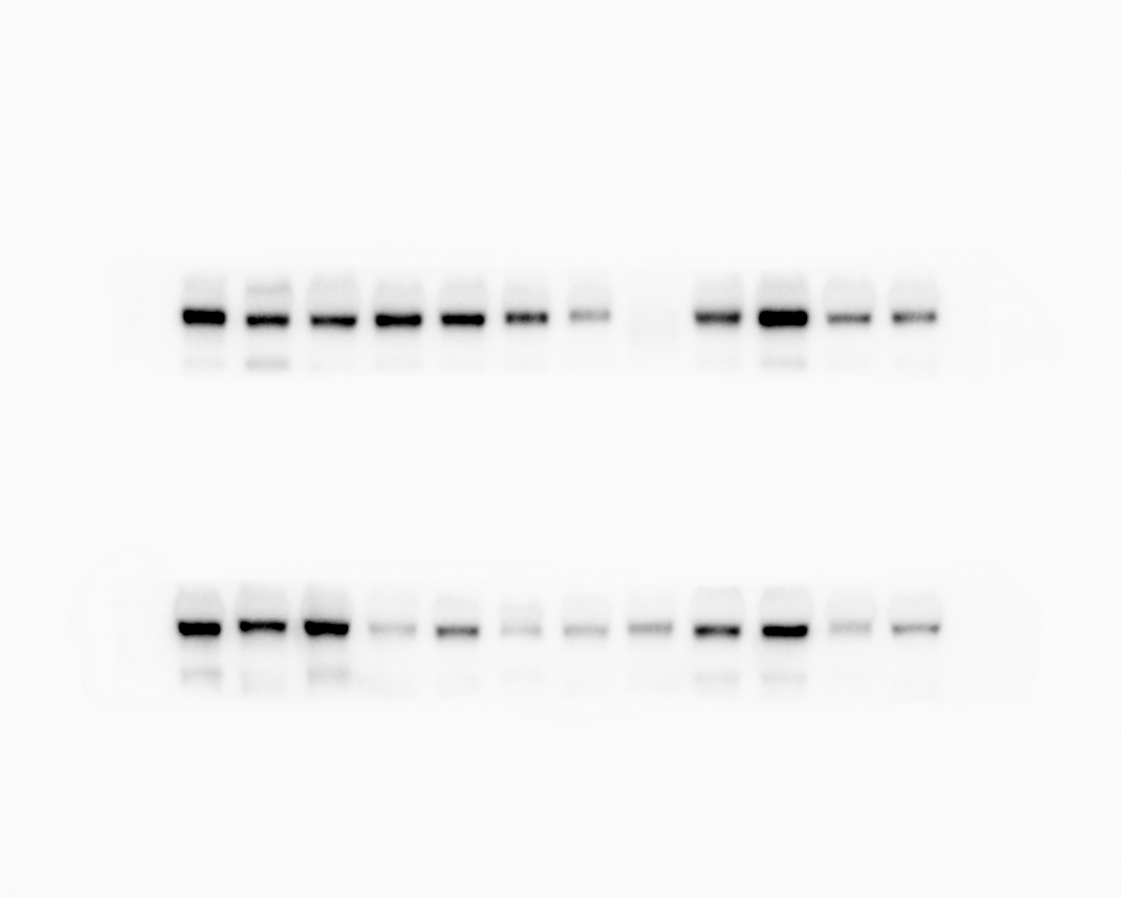

Supplement: Supplemental Information 1 [file peerj-10-13867-s001.zip › uncropped blots in JPEG/figure 3/figure 3A/B6 mice_CQ_24hr_AT_p62_upper_2019-04-16.jpg]

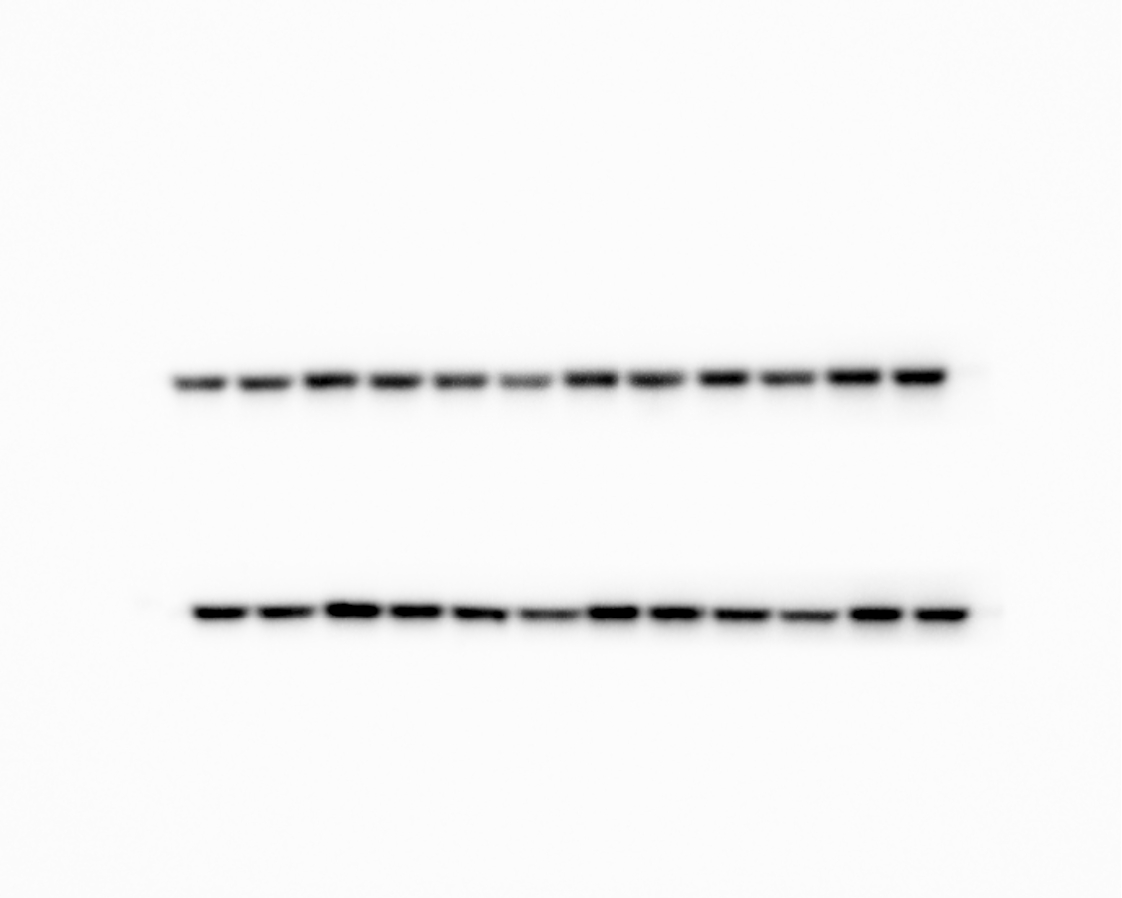

Supplement: Supplemental Information 1 [file peerj-10-13867-s001.zip › uncropped blots in JPEG/figure 4/figure 4A/3T3-L1_CQ_+-Ins_Akt_upper_2018-08-10.jpg]

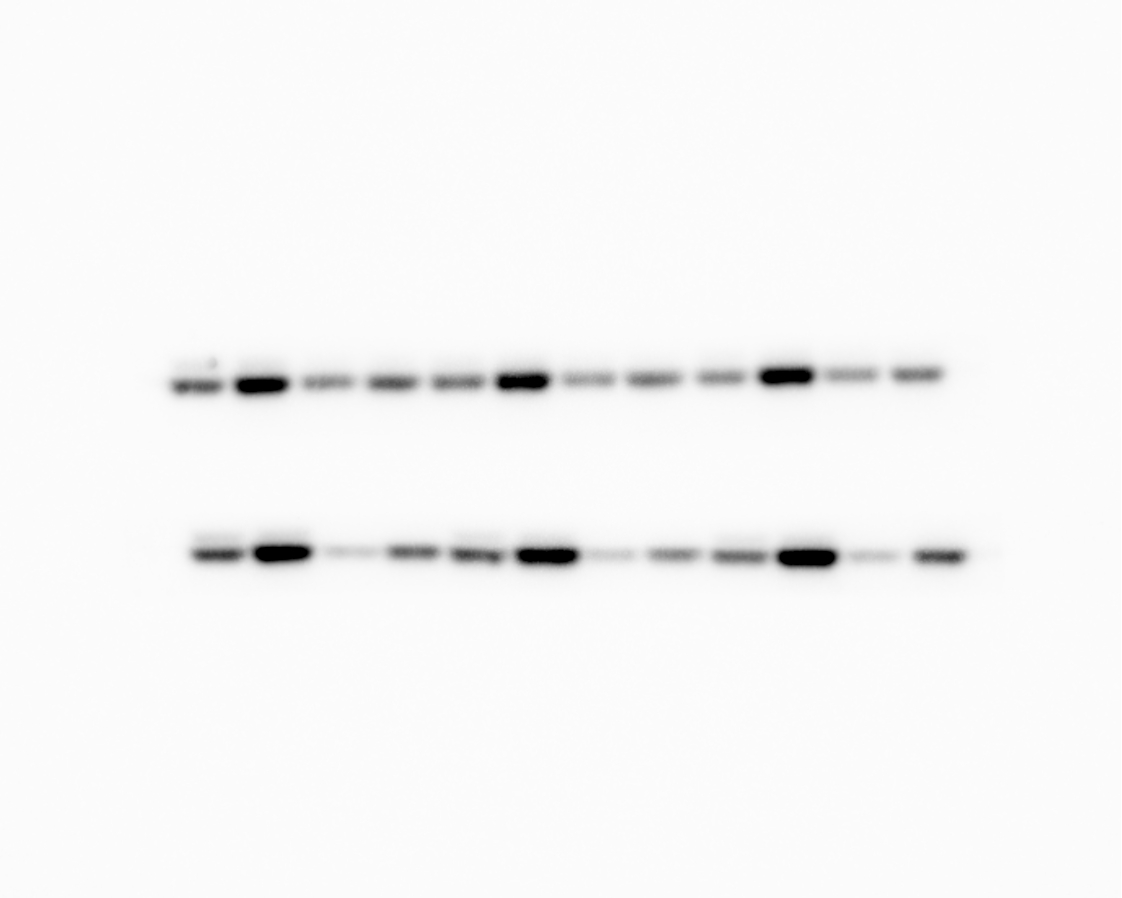

Supplement: Supplemental Information 1 [file peerj-10-13867-s001.zip › uncropped blots in JPEG/figure 4/figure 4A/3T3-L1_CQ_+-Ins_p-Akt_upper_2018-08-08.jpg]

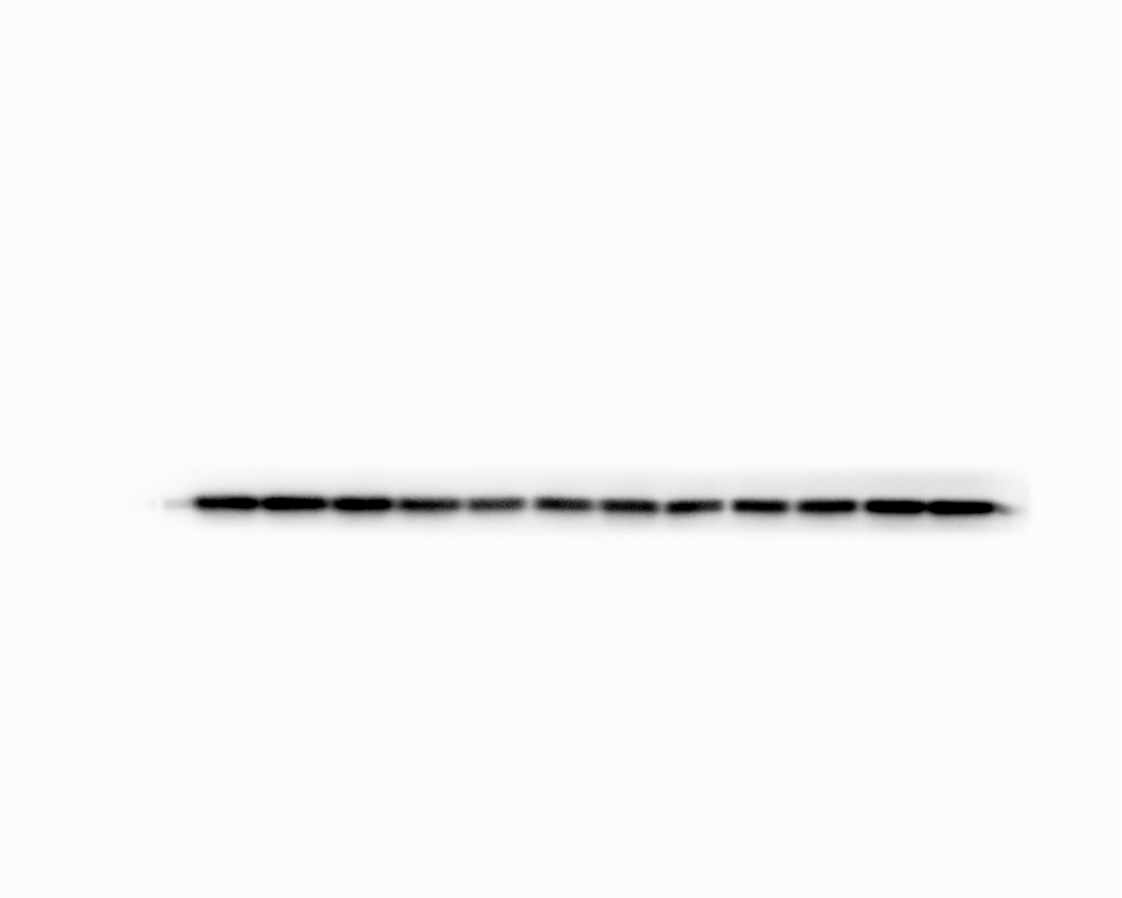

Supplement: Supplemental Information 1 [file peerj-10-13867-s001.zip › uncropped blots in JPEG/figure 4/figure 4A/3T3-L1_CQ_GAPDH for LC3_2018-08-10.jpg]

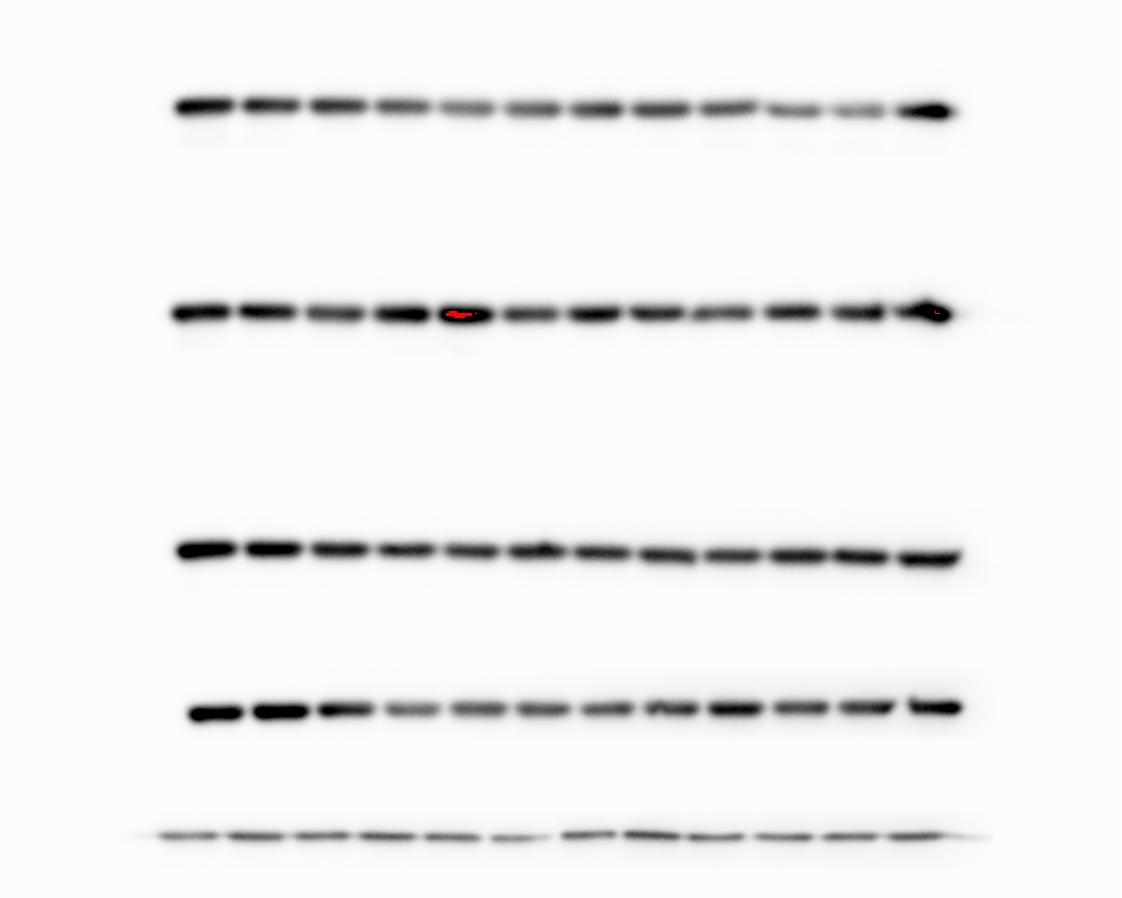

Supplement: Supplemental Information 1 [file peerj-10-13867-s001.zip › uncropped blots in JPEG/figure 4/figure 4A/3T3-L1_CQ_GAPDH for p62_3rd_2018-08-04.jpg]

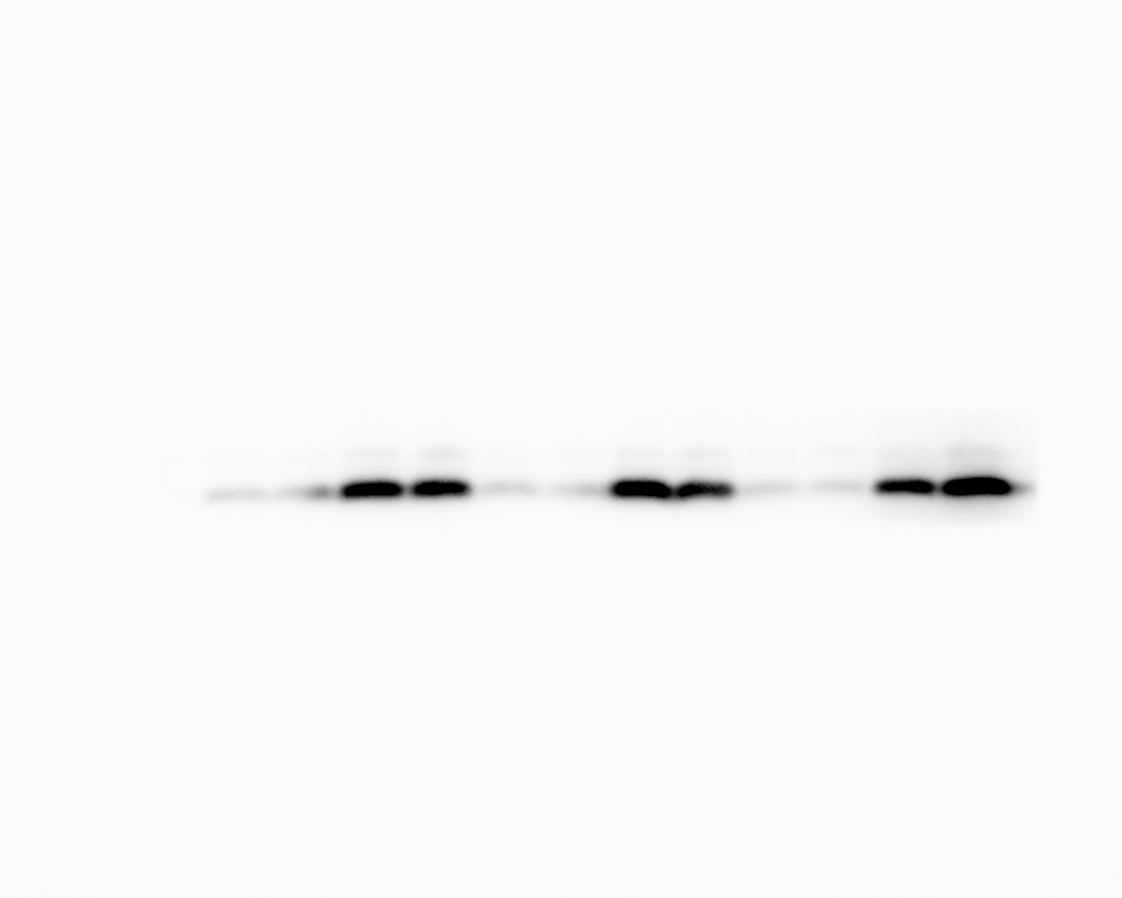

Supplement: Supplemental Information 1 [file peerj-10-13867-s001.zip › uncropped blots in JPEG/figure 4/figure 4A/3T3-L1_CQ_LC3_2018-08-10.jpg]

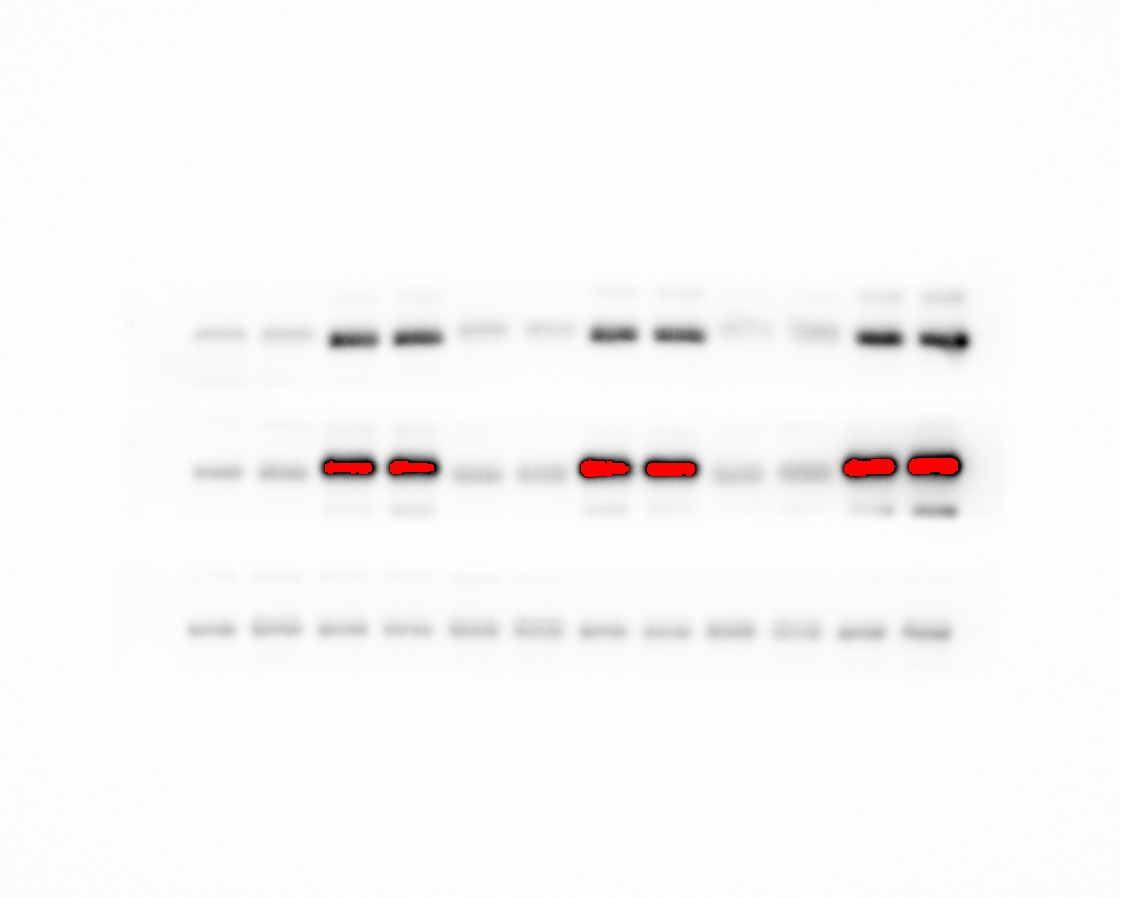

Supplement: Supplemental Information 1 [file peerj-10-13867-s001.zip › uncropped blots in JPEG/figure 4/figure 4A/3T3-L1_CQ_p62_upper_2018-08-08.jpg]

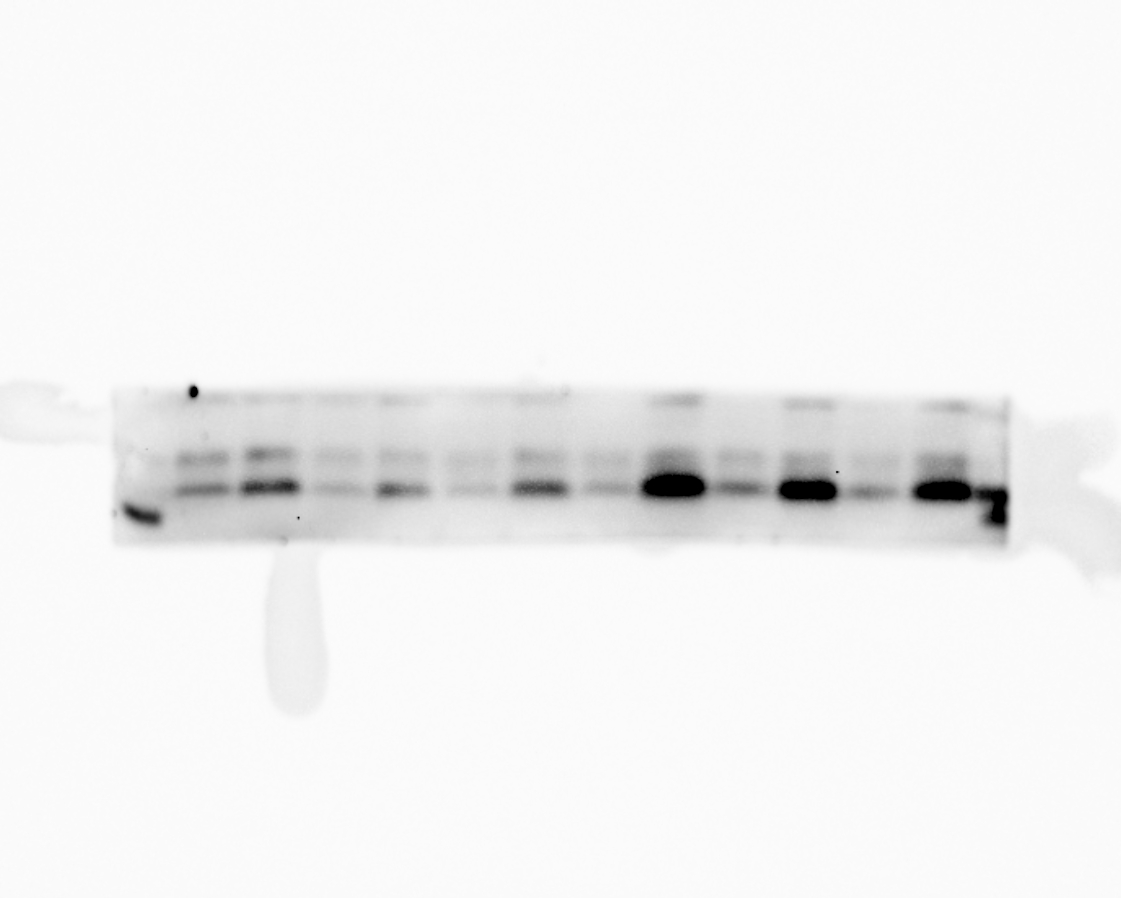

Supplement: Supplemental Information 1 [file peerj-10-13867-s001.zip › uncropped blots in JPEG/figure 4/figure 4E/3T3-L1_CQ_CC3_left_2018-08-29.jpg]

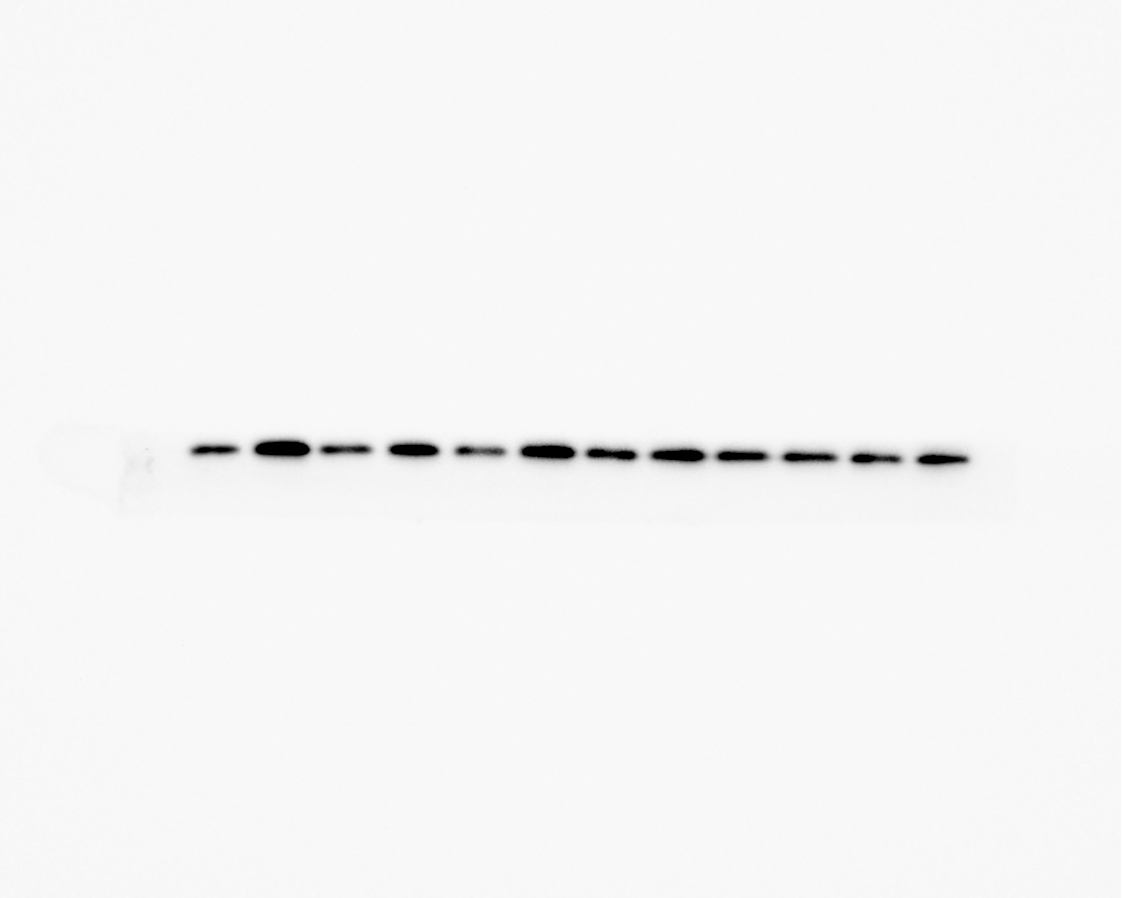

Supplement: Supplemental Information 1 [file peerj-10-13867-s001.zip › uncropped blots in JPEG/figure 4/figure 4E/3T3-L1_CQ_CHOP_left_2018-07-31.jpg]

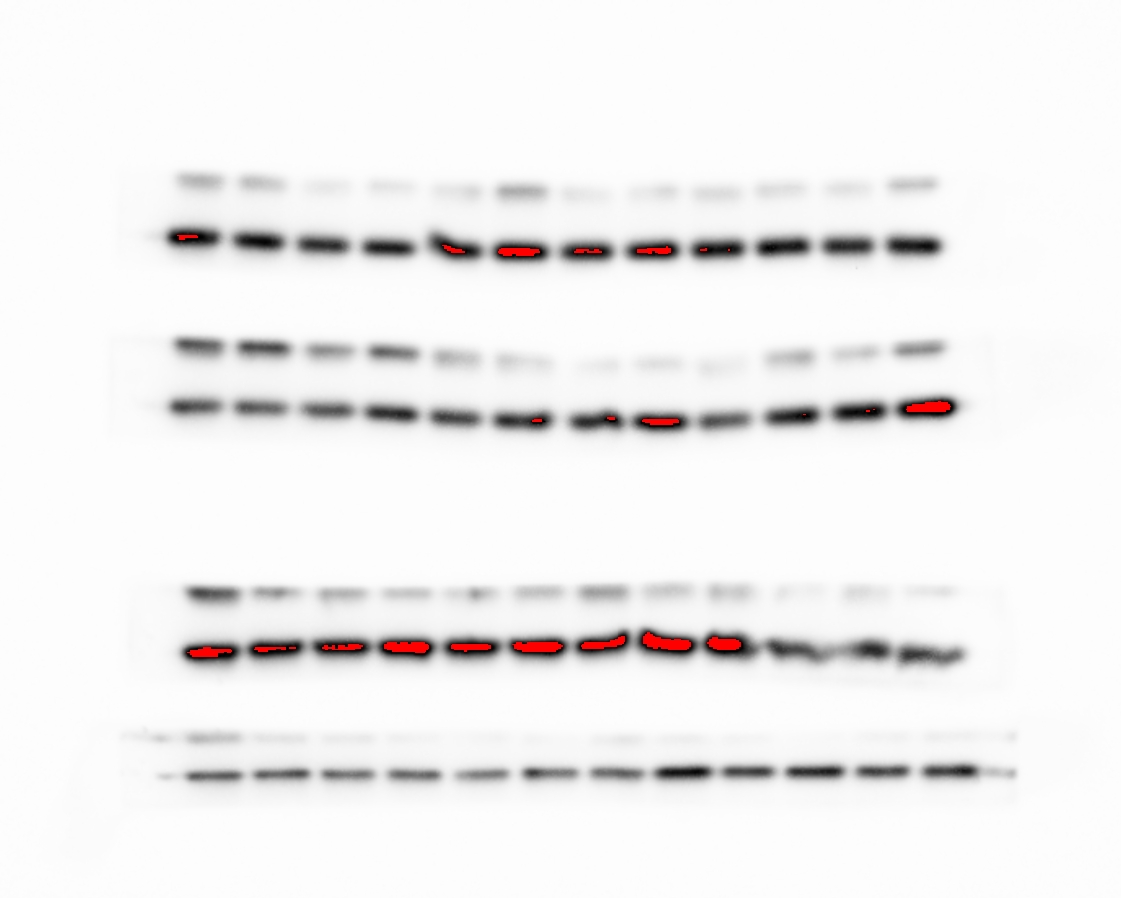

Supplement: Supplemental Information 1 [file peerj-10-13867-s001.zip › uncropped blots in JPEG/figure 4/figure 4E/3T3-L1_CQ_GAPDH for CC3_lower_left_2018-08-29.jpg]

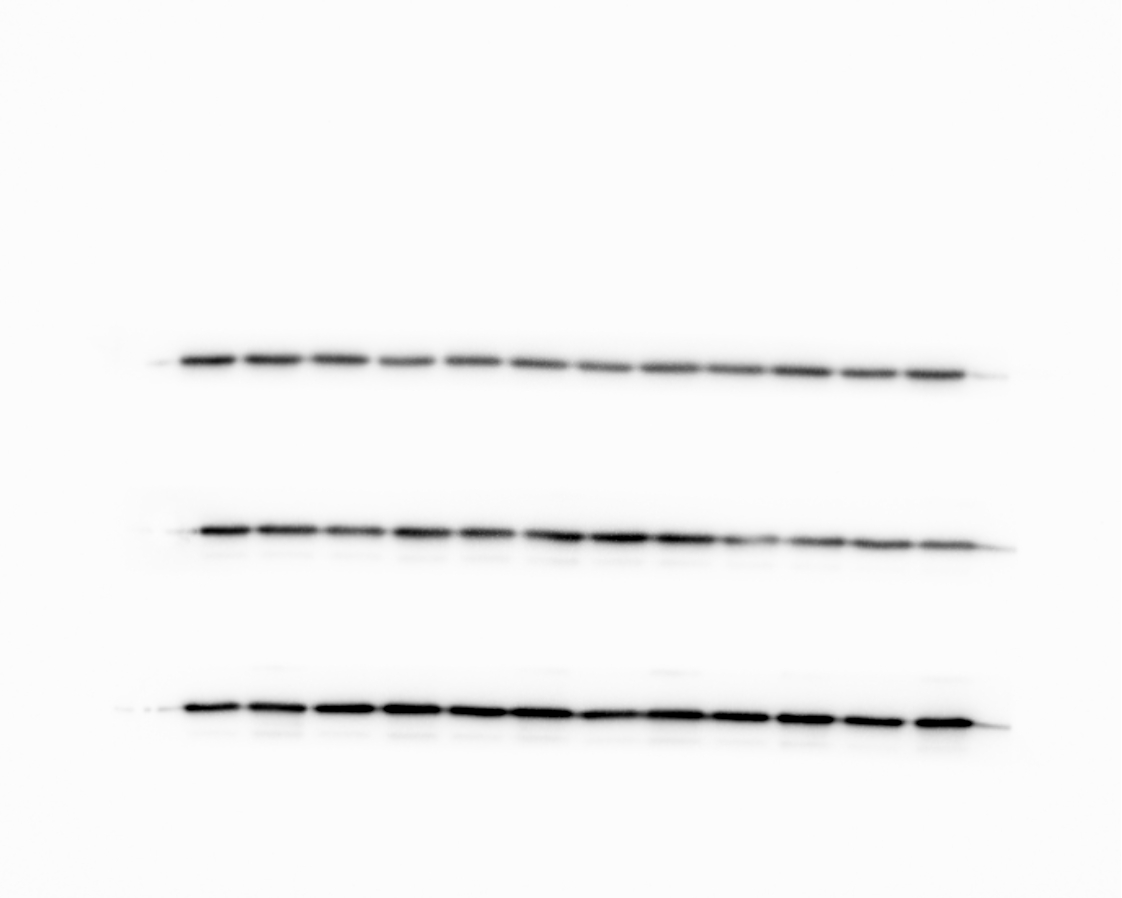

Supplement: Supplemental Information 1 [file peerj-10-13867-s001.zip › uncropped blots in JPEG/figure 4/figure 4E/3T3-L1_CQ_GAPDH for CHOP_left_2018-07-31.jpg]

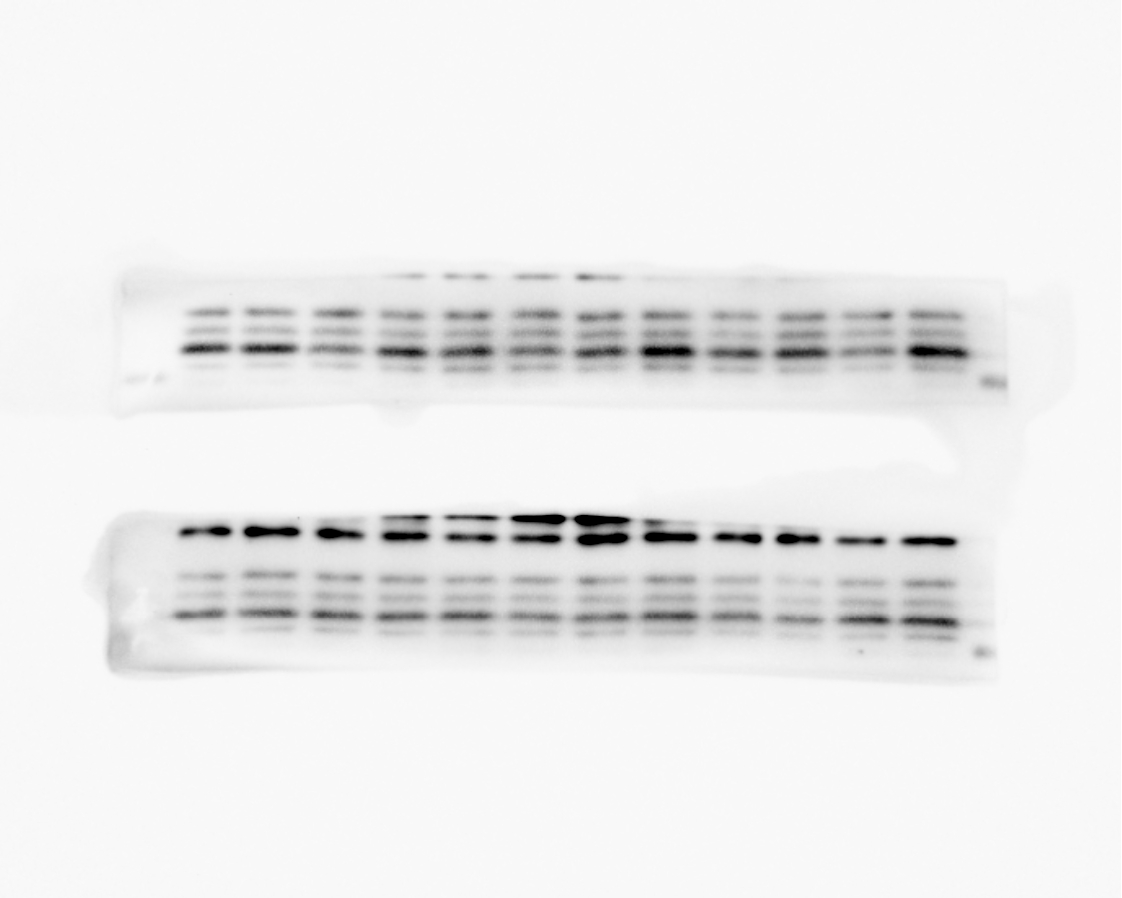

Supplement: Supplemental Information 1 [file peerj-10-13867-s001.zip › uncropped blots in JPEG/figure 5/figure 5A/3T3-L1_CQ_Dose_CC3_upper_2019-04-01.jpg]

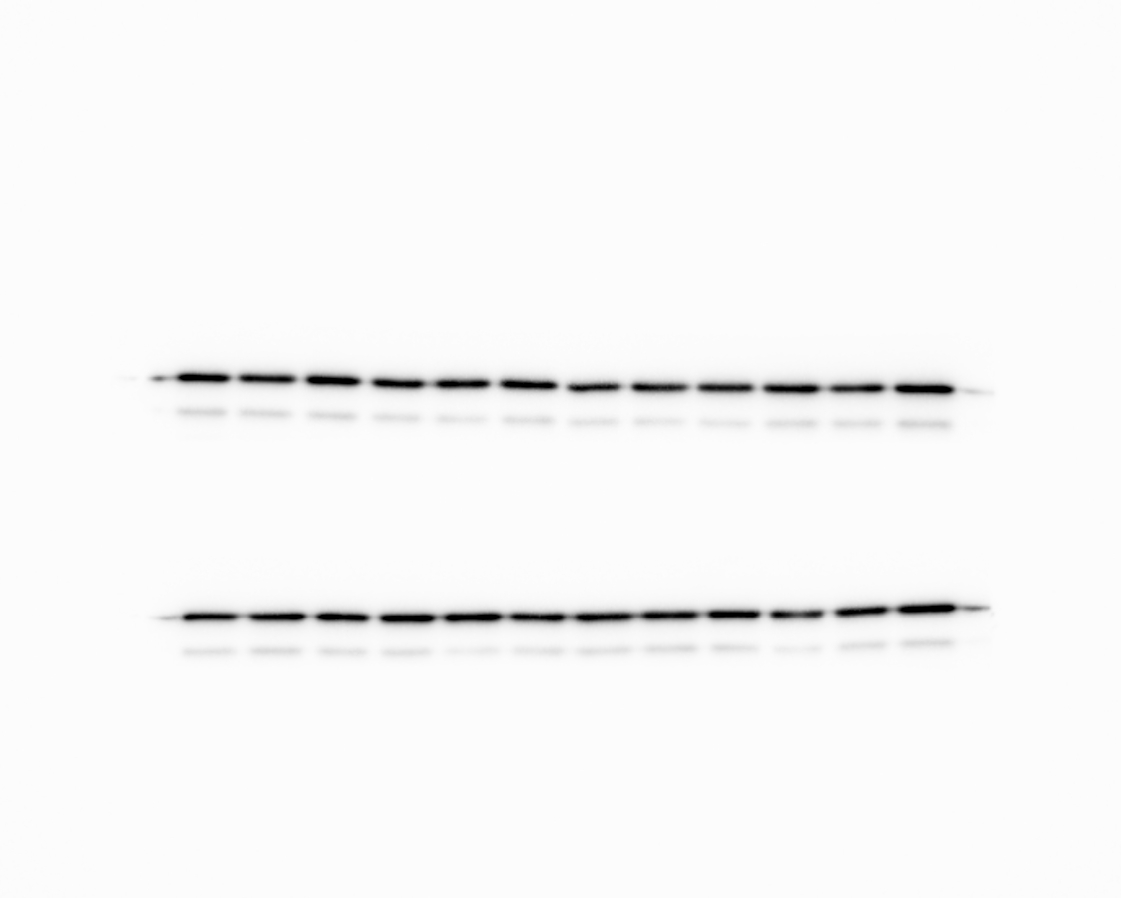

Supplement: Supplemental Information 1 [file peerj-10-13867-s001.zip › uncropped blots in JPEG/figure 5/figure 5A/3T3-L1_CQ_Dose_GAPDH for CC3_upper_2019-04-01.jpg]

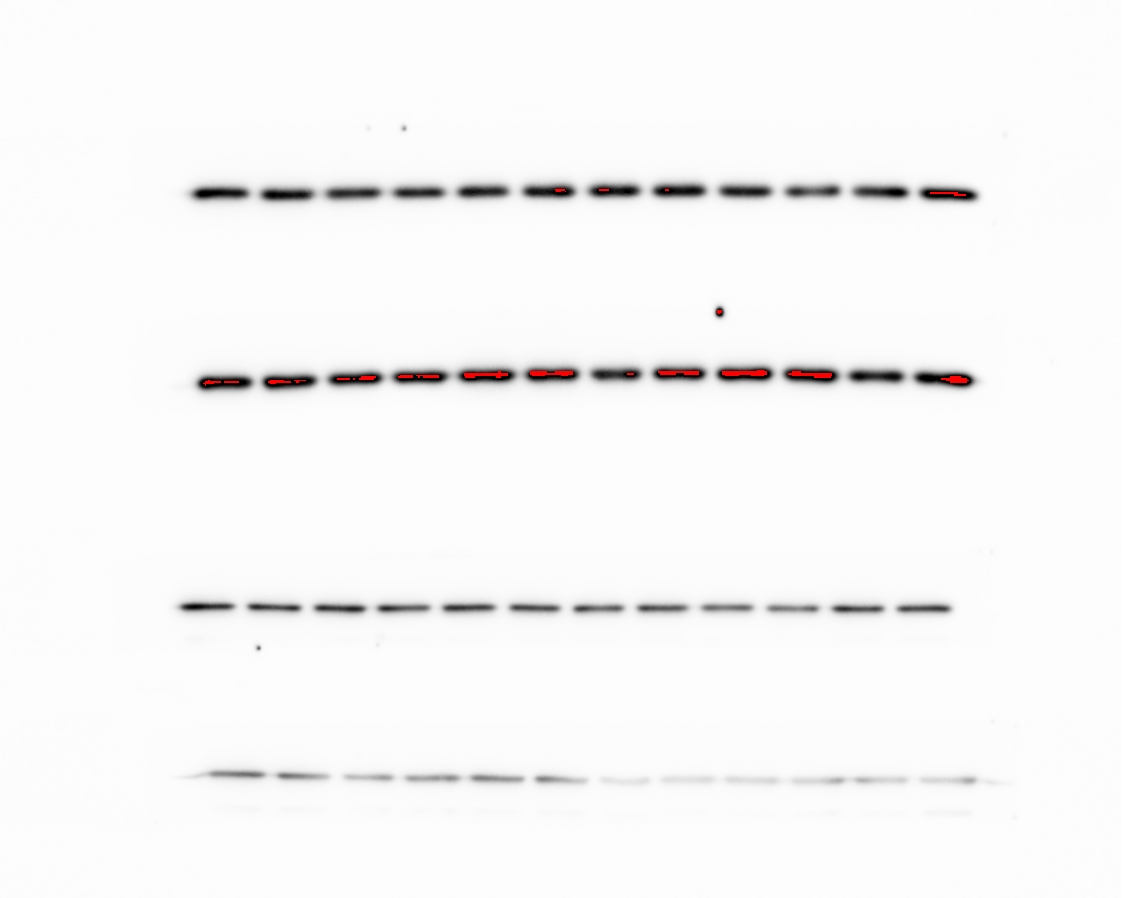

Supplement: Supplemental Information 1 [file peerj-10-13867-s001.zip › uncropped blots in JPEG/figure 5/figure 5A/3T3-L1_CQ_Dose_GAPDH for LC3_3rd_2019-05-02.jpg]

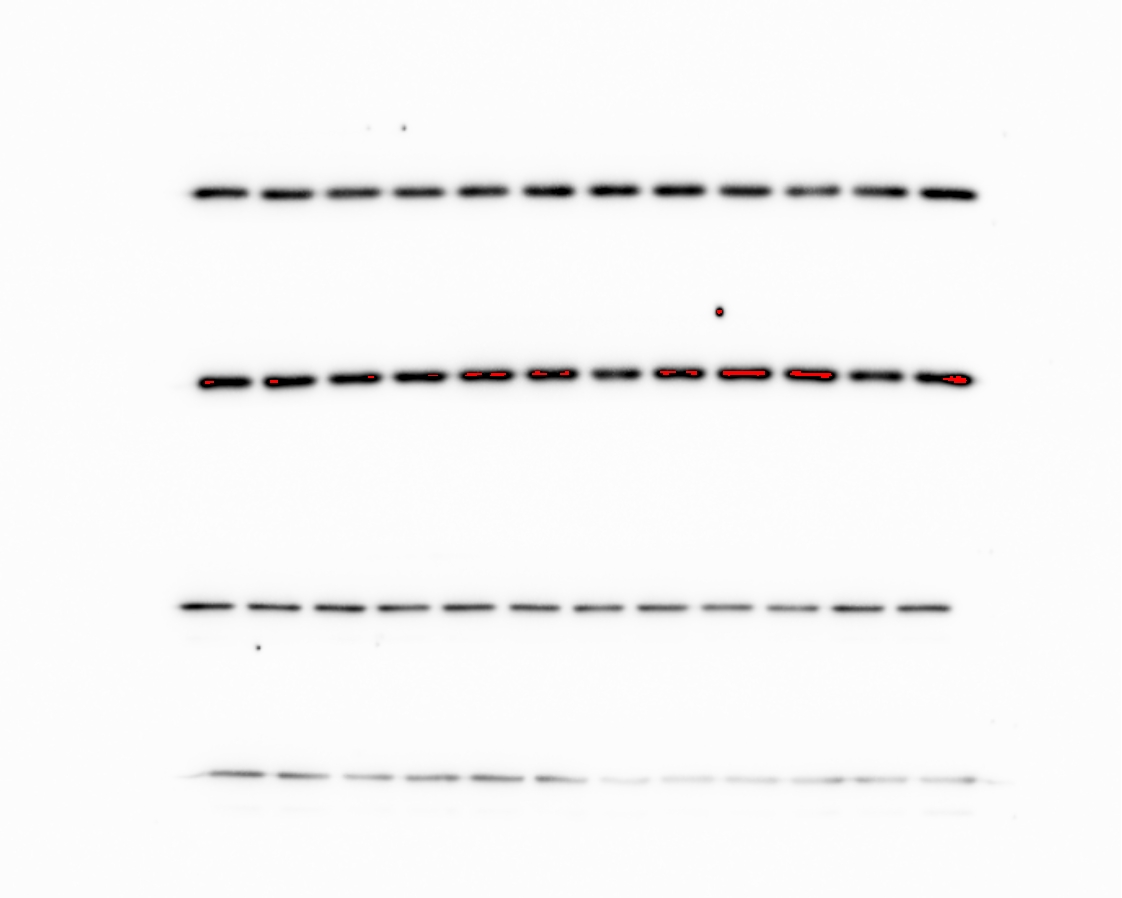

Supplement: Supplemental Information 1 [file peerj-10-13867-s001.zip › uncropped blots in JPEG/figure 5/figure 5A/3T3-L1_CQ_Dose_GAPDH for p62_upper_2019-05-02.jpg]

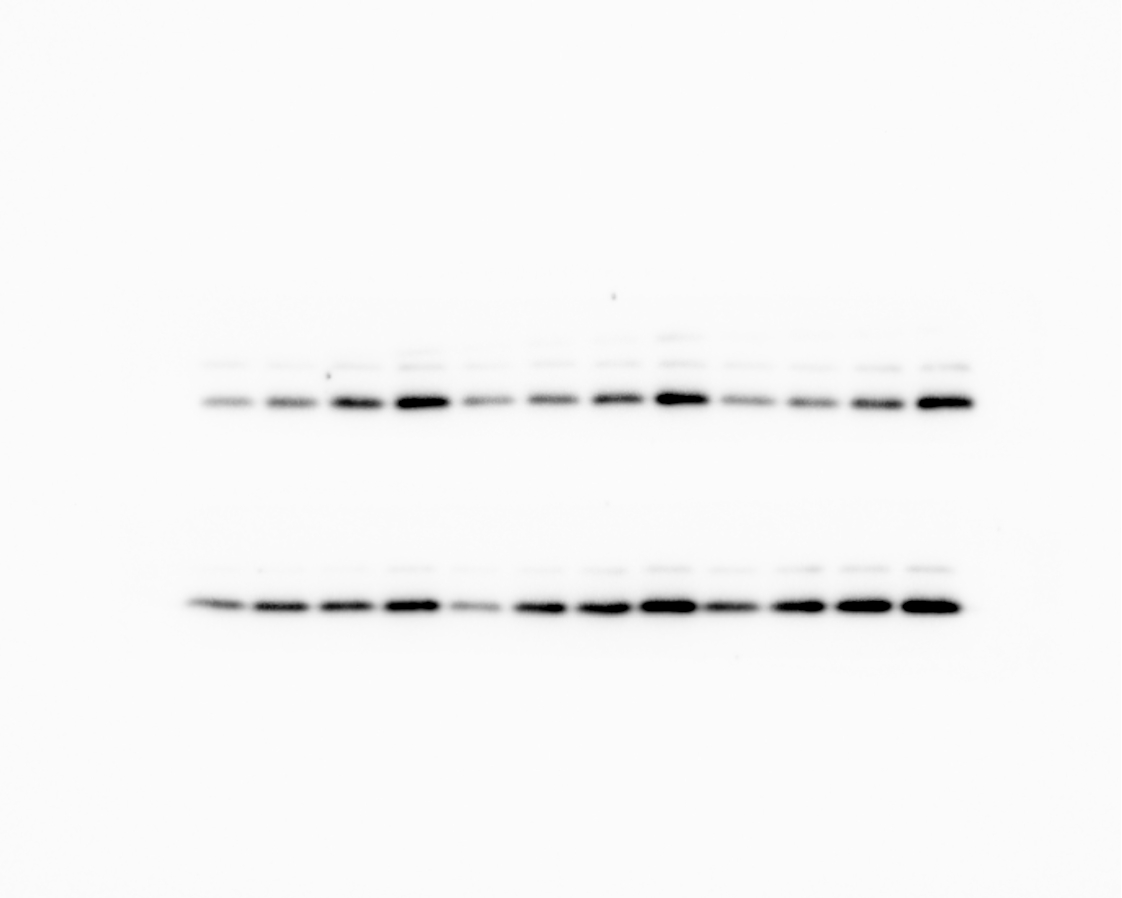

Supplement: Supplemental Information 1 [file peerj-10-13867-s001.zip › uncropped blots in JPEG/figure 5/figure 5A/3T3-L1_CQ_Dose_LC3_upper_2019-05-02.jpg]

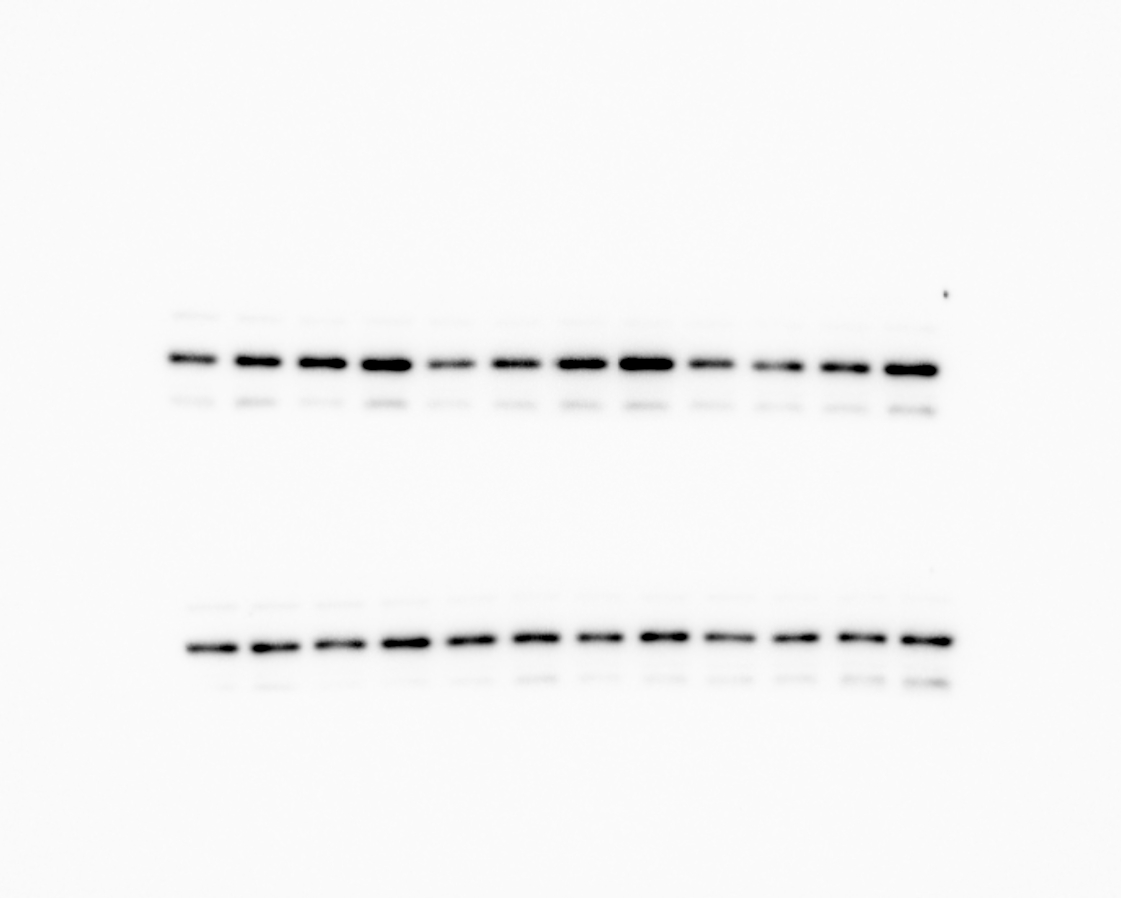

Supplement: Supplemental Information 1 [file peerj-10-13867-s001.zip › uncropped blots in JPEG/figure 5/figure 5A/3T3-L1_CQ_Dose_p62_upper_2019-05-02.jpg]

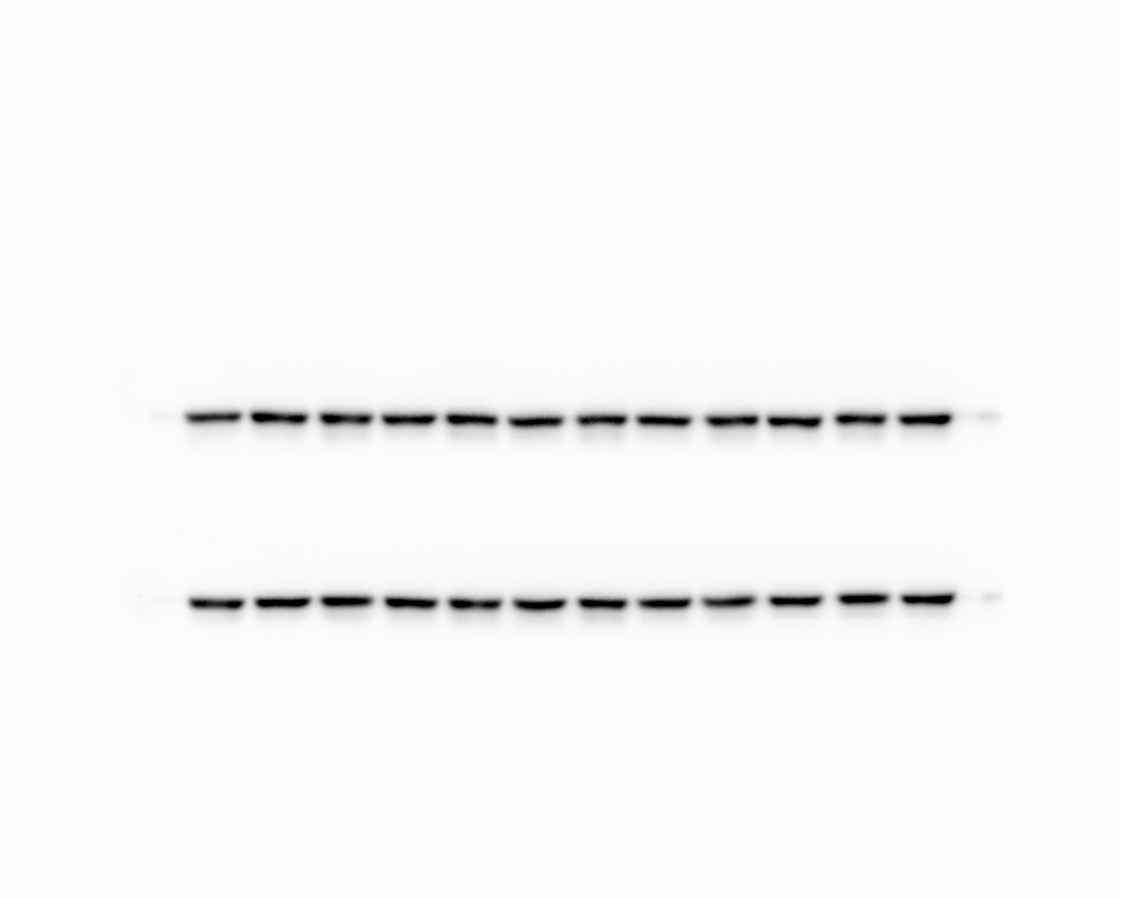

Supplement: Supplemental Information 1 [file peerj-10-13867-s001.zip › uncropped blots in JPEG/figure 5/figure 5E/3T3-L1_CQ_24hr_+-Ins_Akt_upper_2019-03-19.jpg]

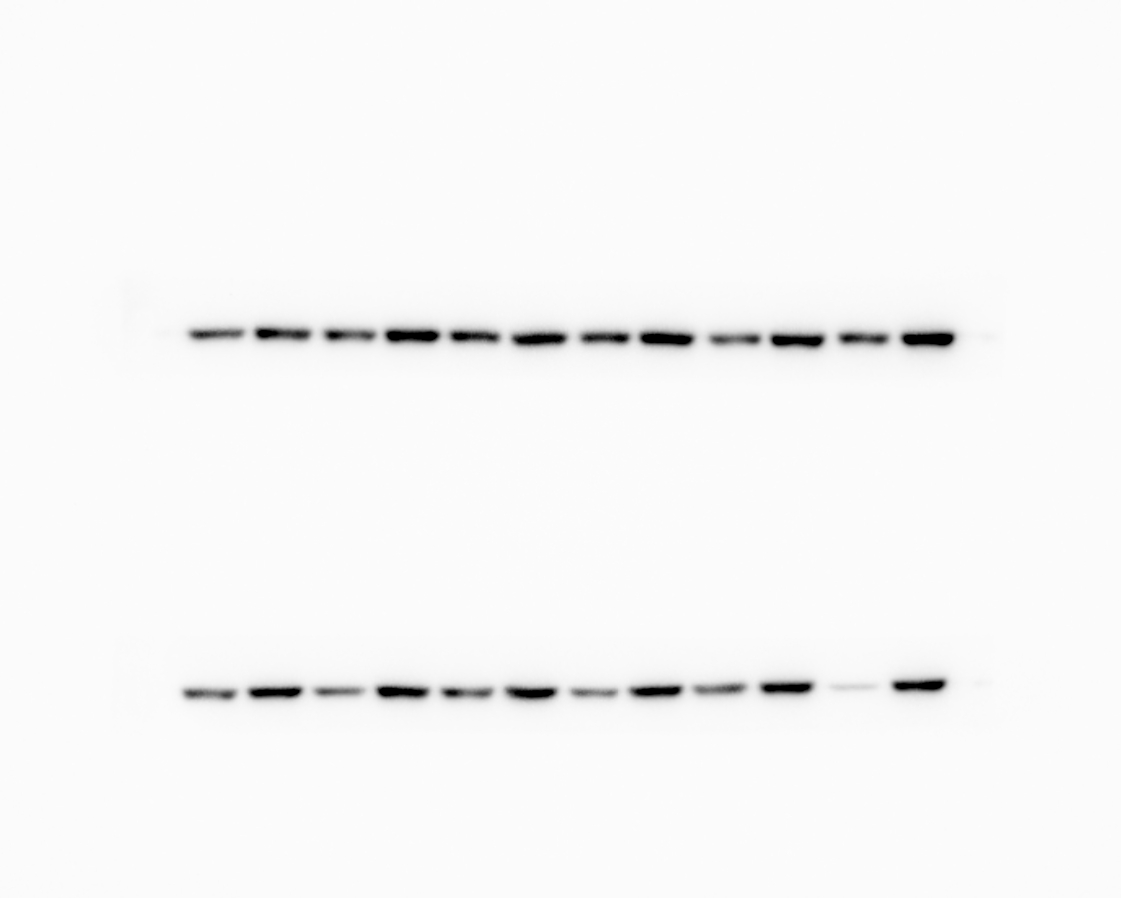

Supplement: Supplemental Information 1 [file peerj-10-13867-s001.zip › uncropped blots in JPEG/figure 5/figure 5E/3T3-L1_CQ_24hr_+-Ins_p-Akt_upper_2019-03-18.jpg]

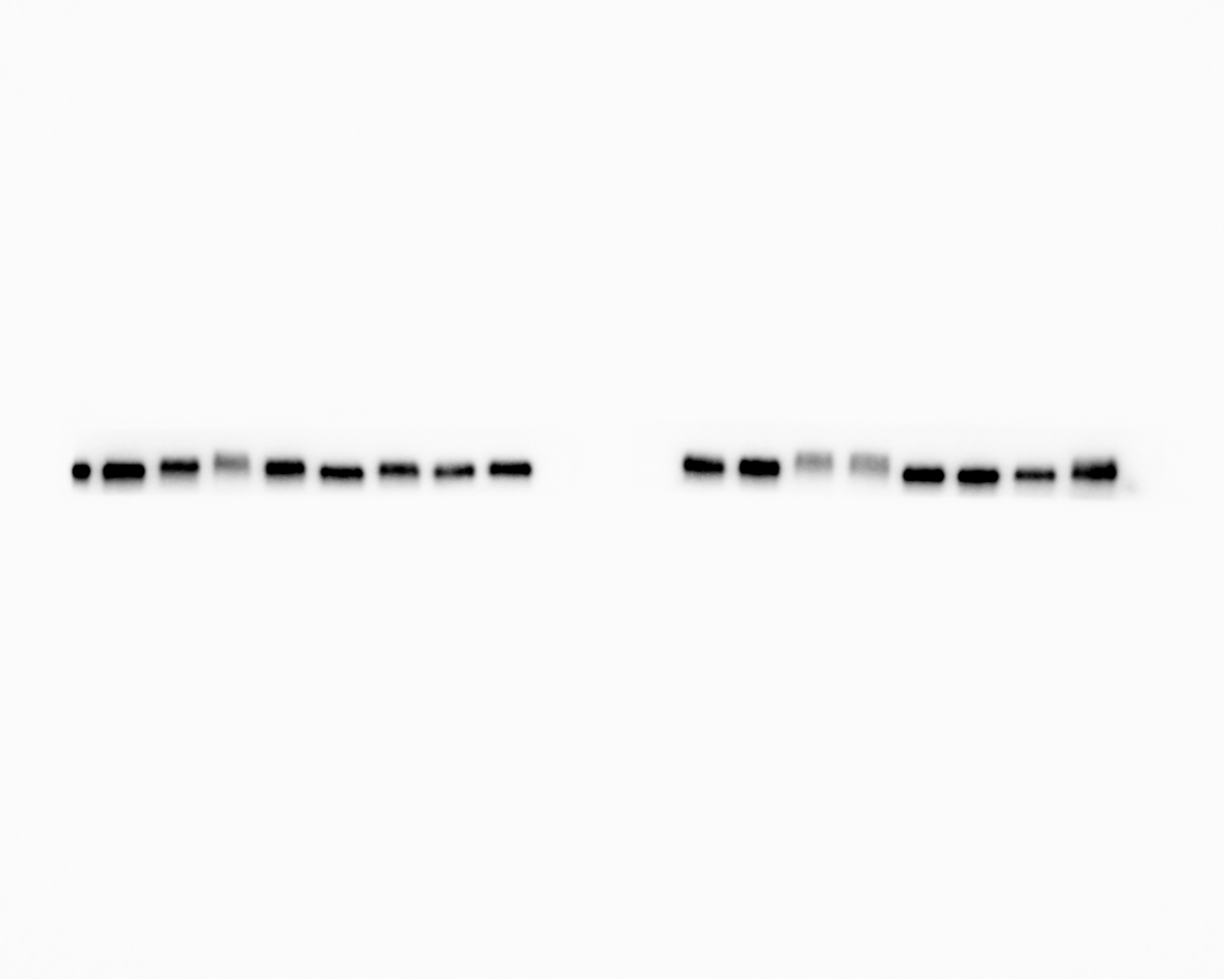

Supplement: Supplemental Information 1 [file peerj-10-13867-s001.zip › uncropped blots in JPEG/figure 6/figure 6A/B6 mice_CQ_10hr_AT_Akt_2019-02-27.jpg]

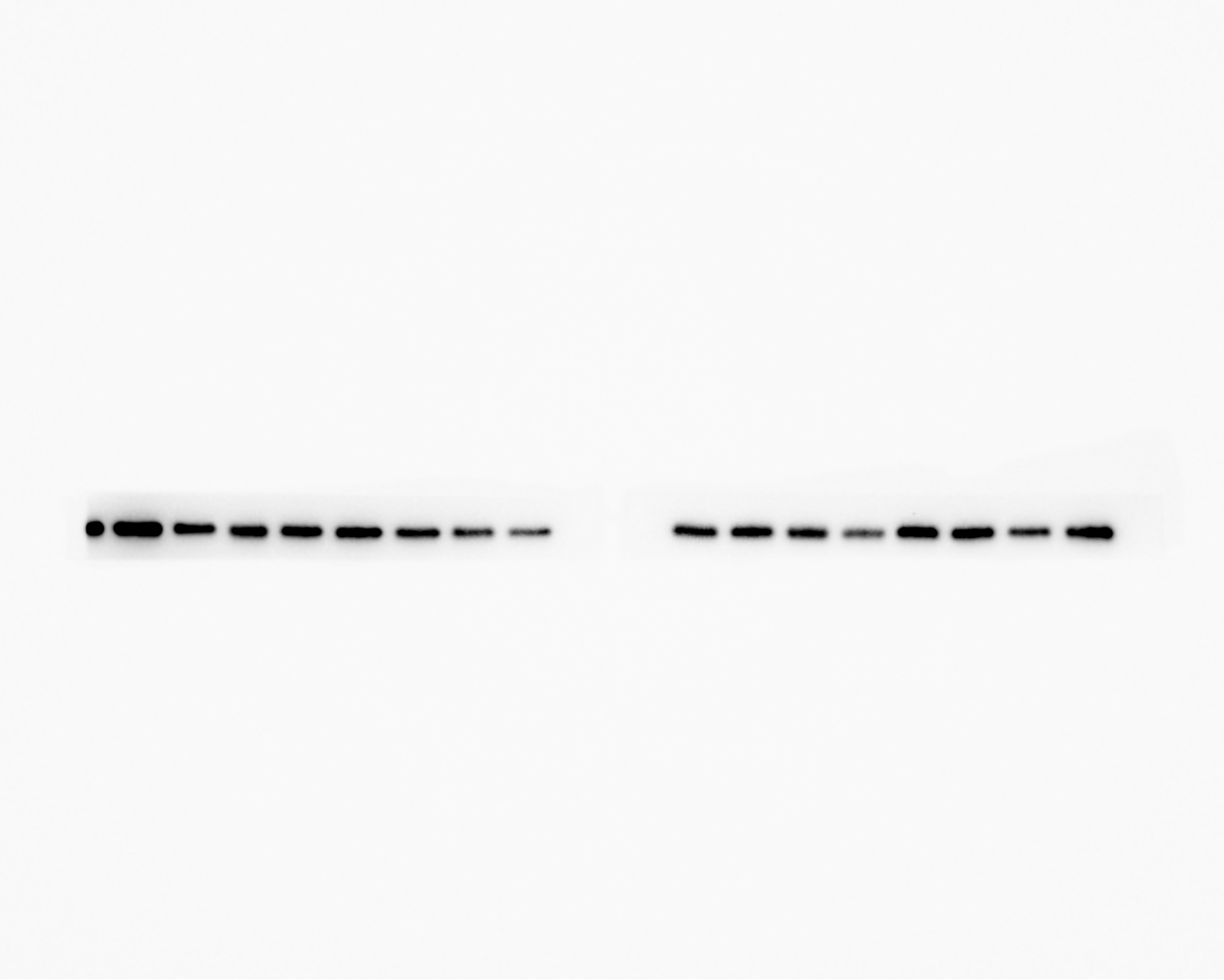

Supplement: Supplemental Information 1 [file peerj-10-13867-s001.zip › uncropped blots in JPEG/figure 6/figure 6A/B6 mice_CQ_10hr_AT_GAPDH for Akt_2019-02-27.jpg]

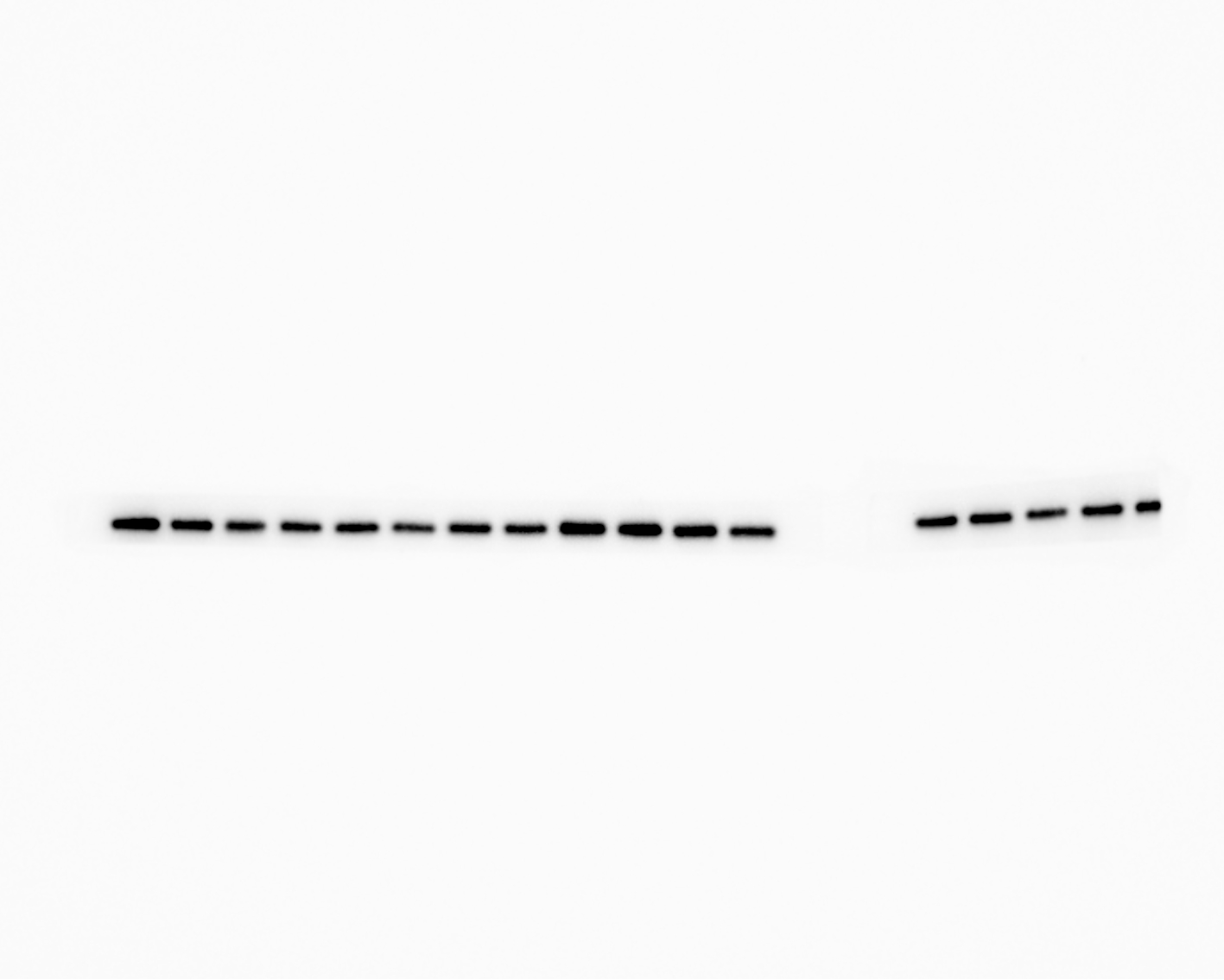

Supplement: Supplemental Information 1 [file peerj-10-13867-s001.zip › uncropped blots in JPEG/figure 6/figure 6A/B6 mice_CQ_10hr_AT_GAPDH for p-Akt_2019-02-27.jpg]

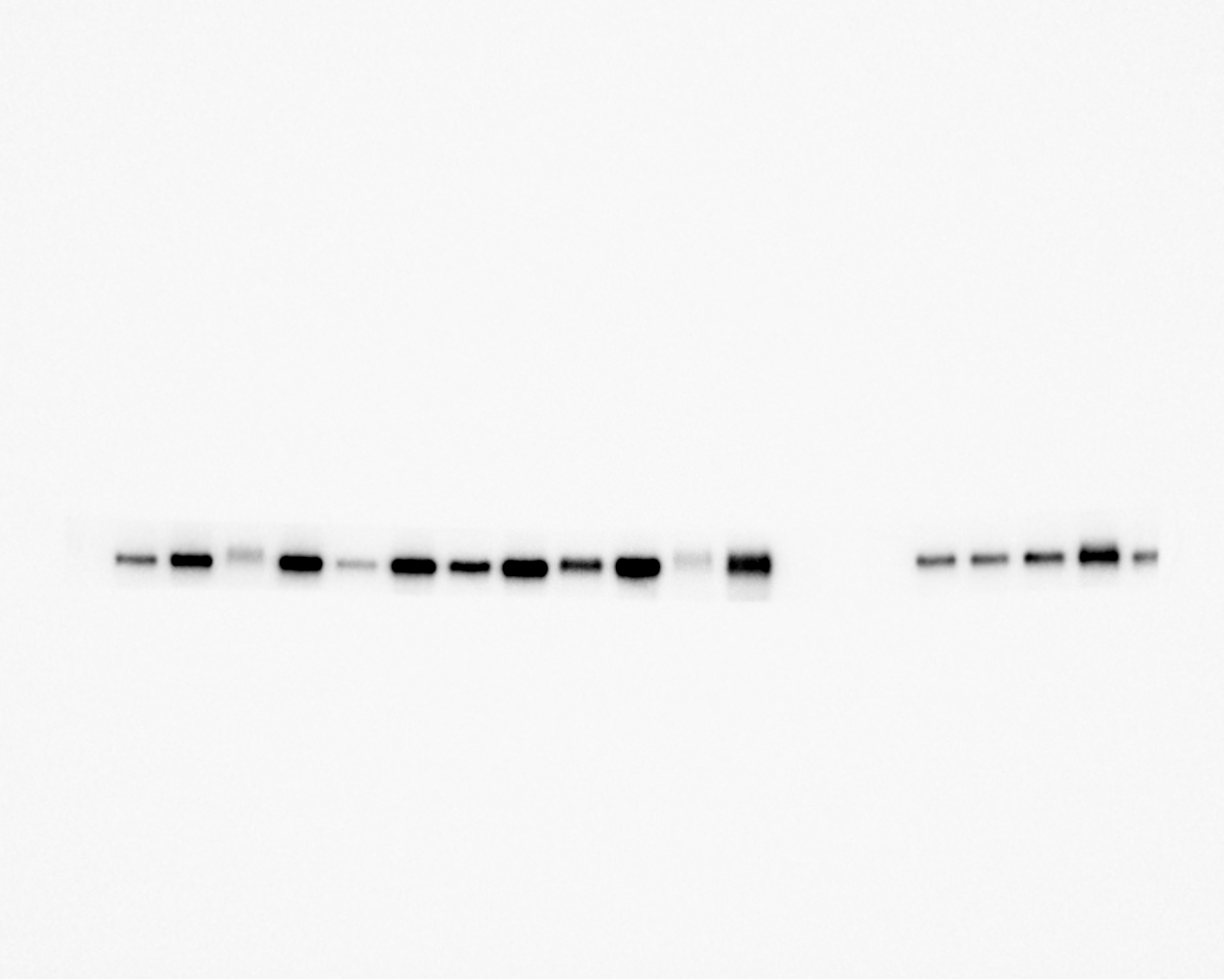

Supplement: Supplemental Information 1 [file peerj-10-13867-s001.zip › uncropped blots in JPEG/figure 6/figure 6A/B6 mice_CQ_10hr_AT_p-Akt_2019-02-27.jpg]

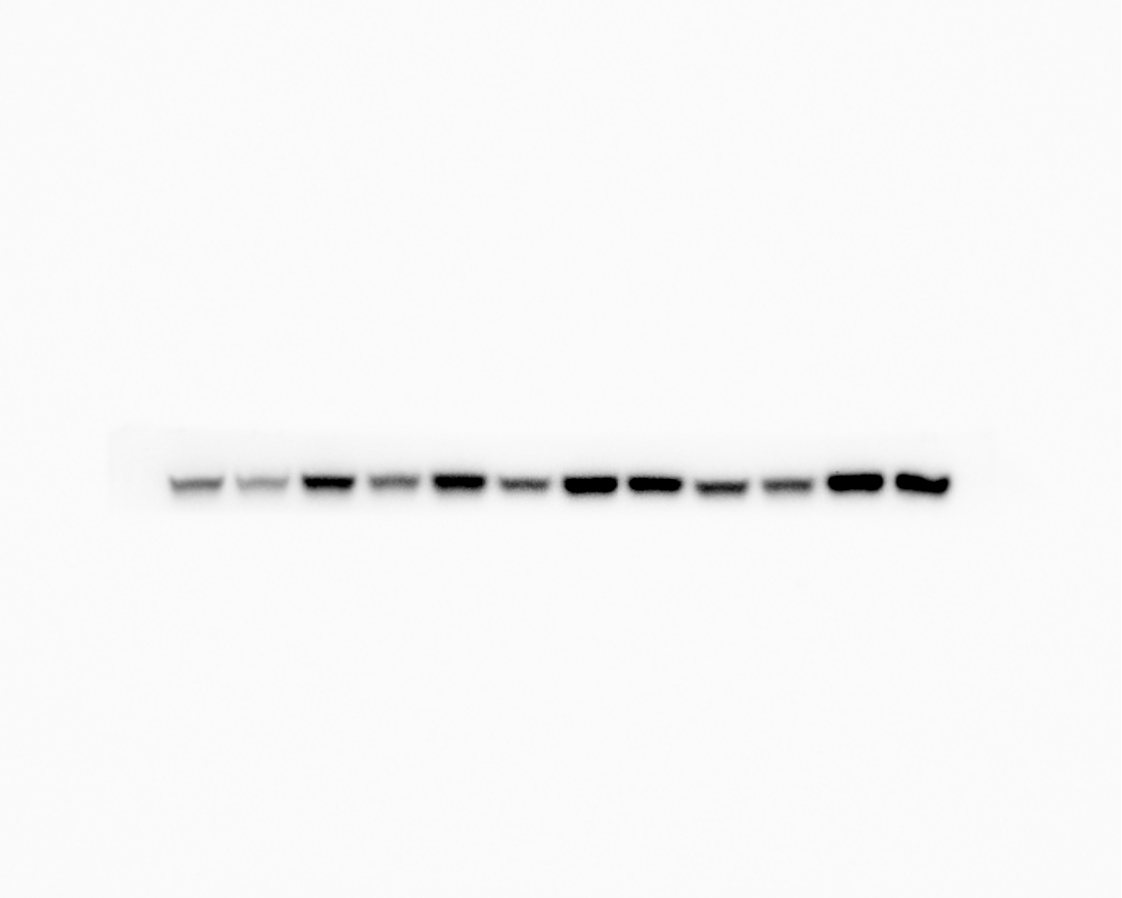

Supplement: Supplemental Information 1 [file peerj-10-13867-s001.zip › uncropped blots in JPEG/figure 6/figure 6C/B6 mice_CQ_24hr_AT_Akt_1_2018-07-06.jpg]

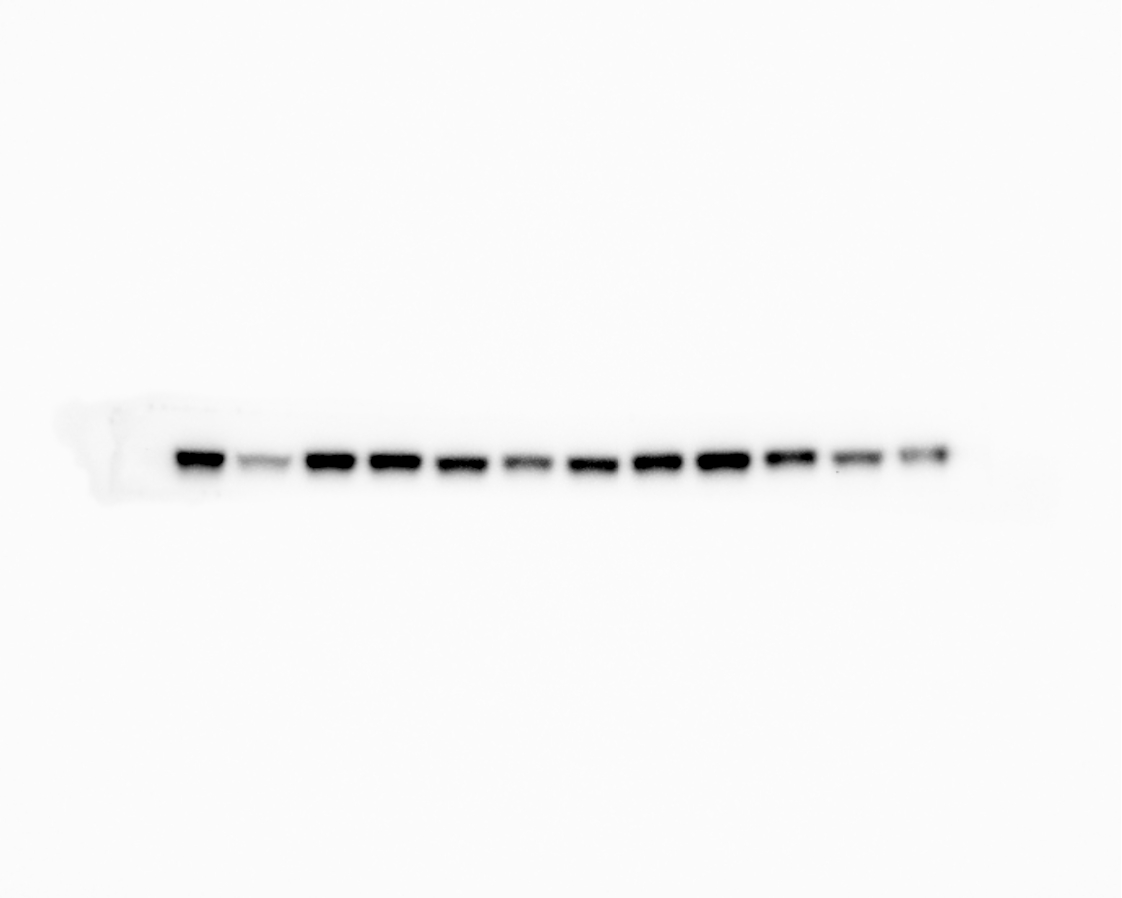

Supplement: Supplemental Information 1 [file peerj-10-13867-s001.zip › uncropped blots in JPEG/figure 6/figure 6C/B6 mice_CQ_24hr_AT_Akt_2_2018-07-06.jpg]

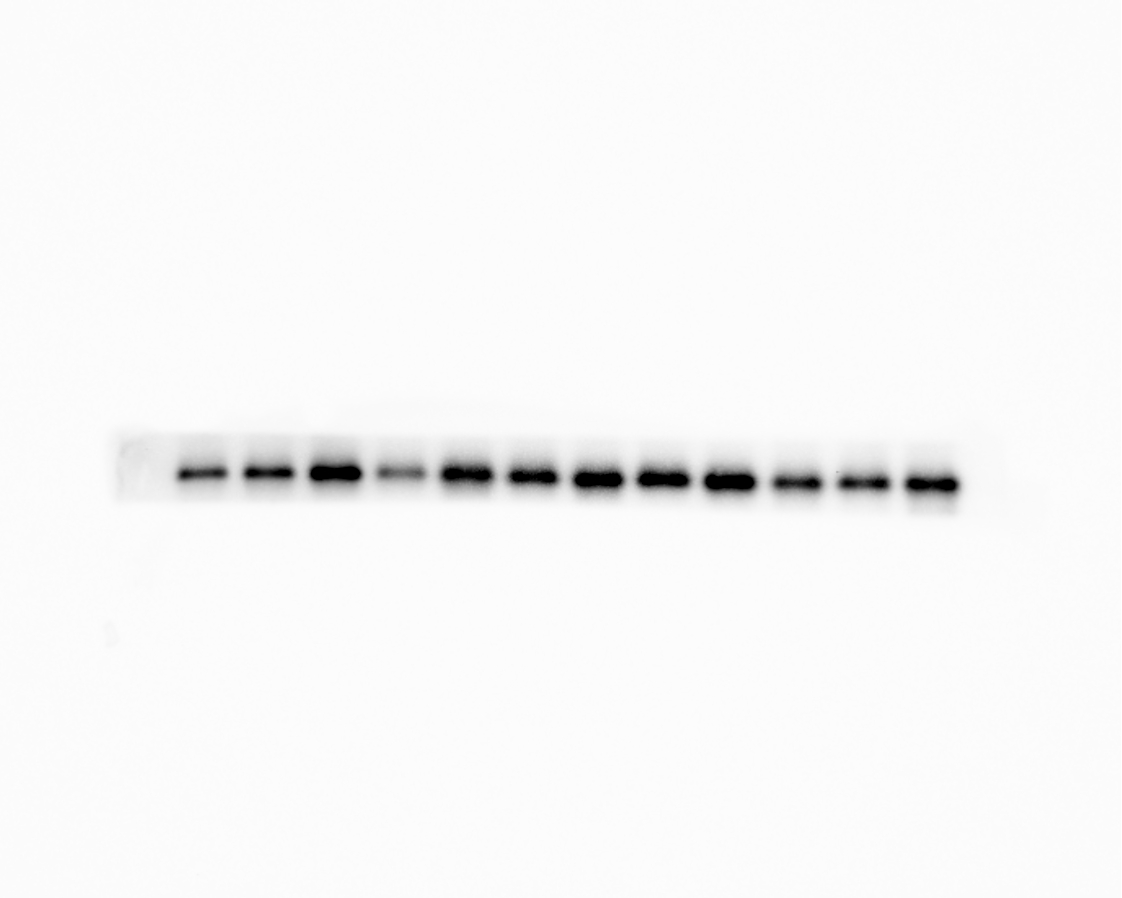

Supplement: Supplemental Information 1 [file peerj-10-13867-s001.zip › uncropped blots in JPEG/figure 6/figure 6C/B6 mice_CQ_24hr_AT_GSK3b_1_2018-07-06.jpg]

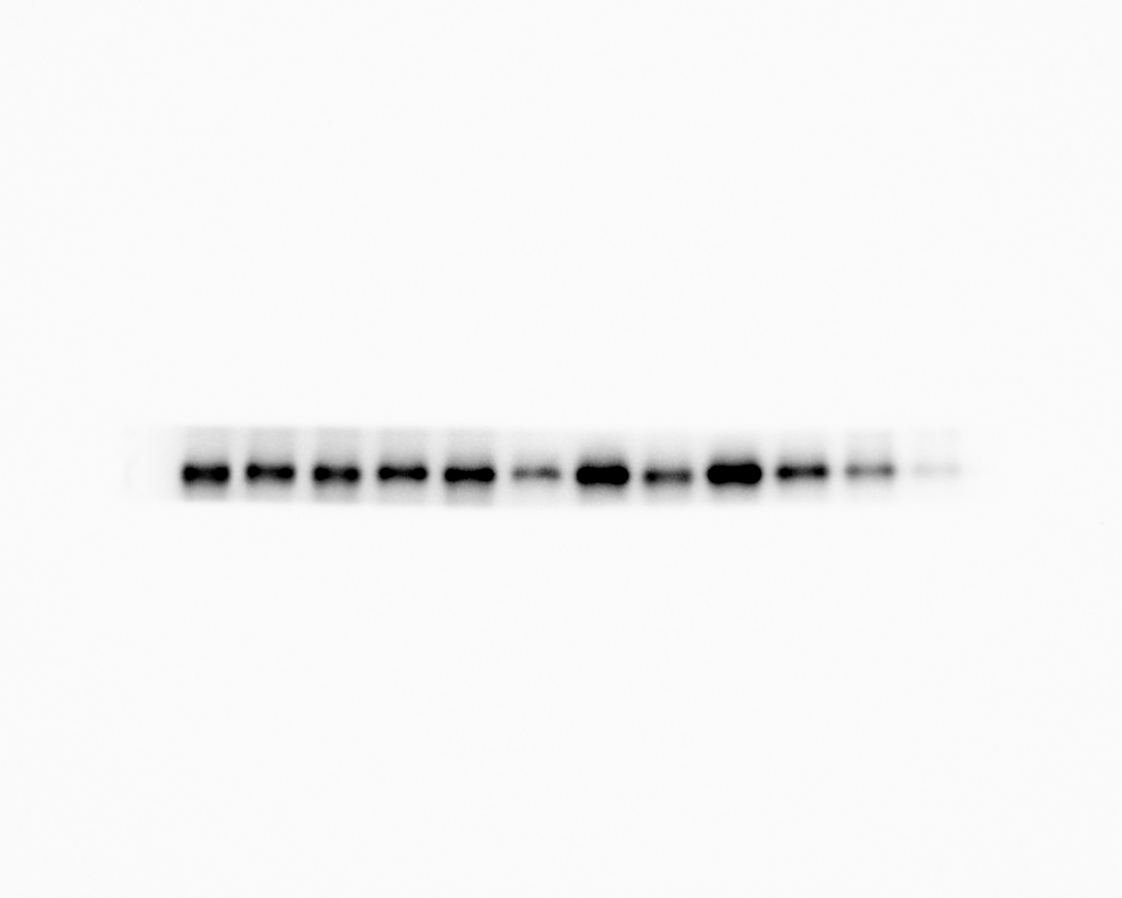

Supplement: Supplemental Information 1 [file peerj-10-13867-s001.zip › uncropped blots in JPEG/figure 6/figure 6C/B6 mice_CQ_24hr_AT_GSK3b_2_2018-07-06.jpg]

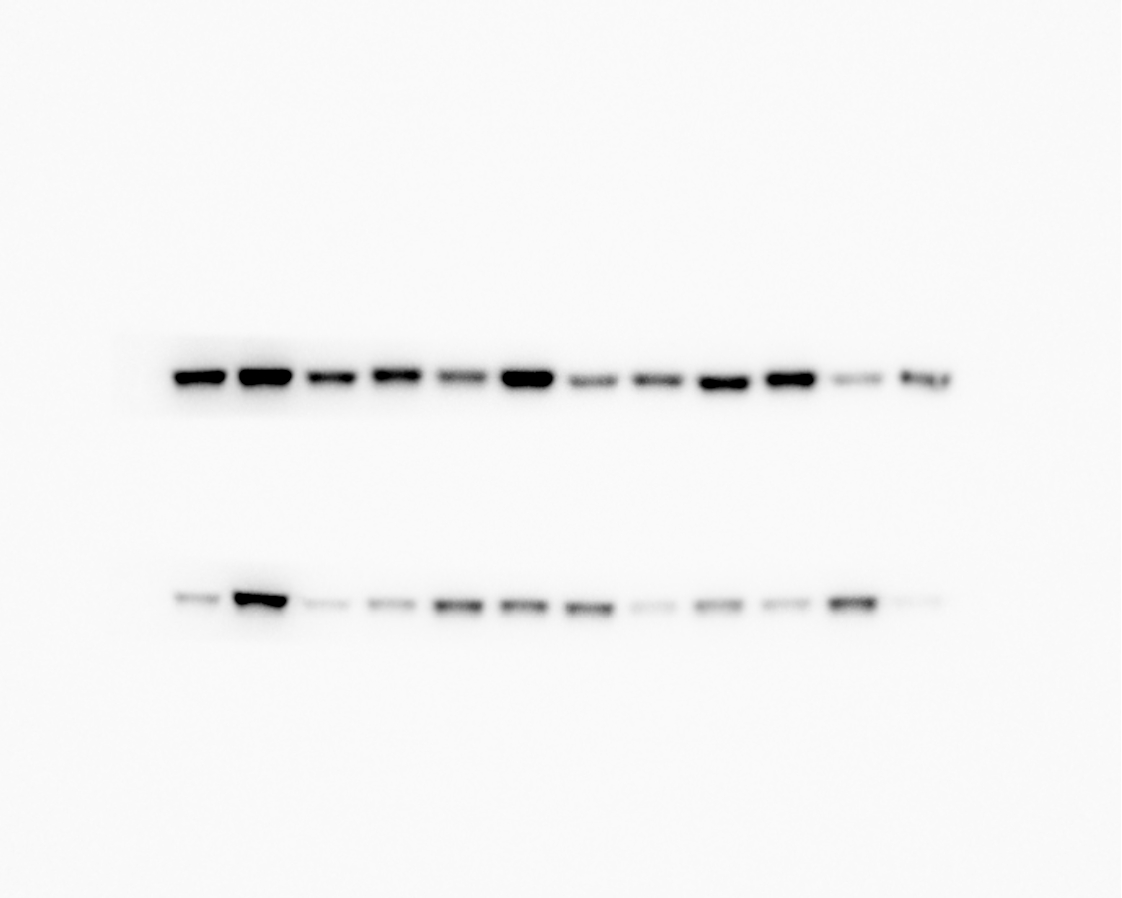

Supplement: Supplemental Information 1 [file peerj-10-13867-s001.zip › uncropped blots in JPEG/figure 6/figure 6C/B6 mice_CQ_24hr_AT_p-Akt_1_upper_2018-07-05.jpg]

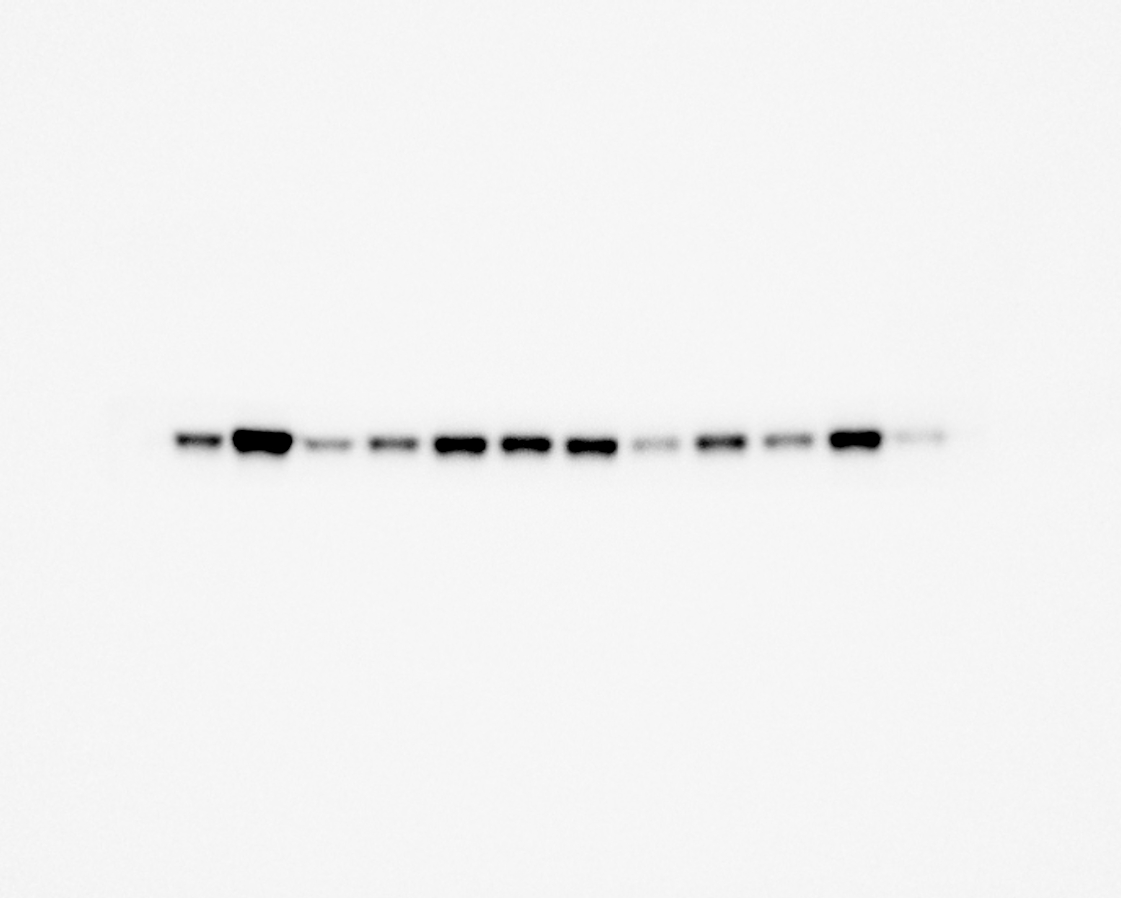

Supplement: Supplemental Information 1 [file peerj-10-13867-s001.zip › uncropped blots in JPEG/figure 6/figure 6C/B6 mice_CQ_24hr_AT_p-Akt_2_2018-07-05.jpg]

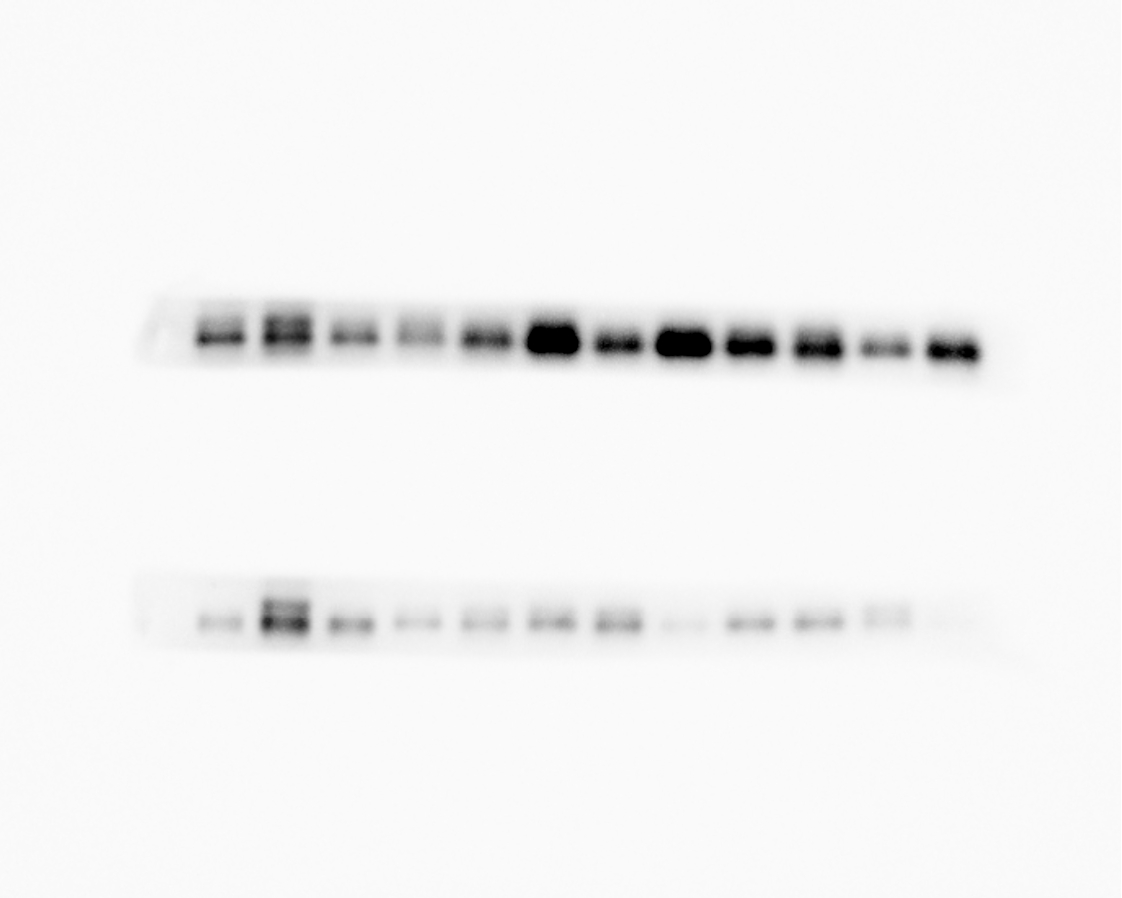

Supplement: Supplemental Information 1 [file peerj-10-13867-s001.zip › uncropped blots in JPEG/figure 6/figure 6C/B6 mice_CQ_24hr_AT_p-GSK3b_1_upper_2018-07-05.jpg]

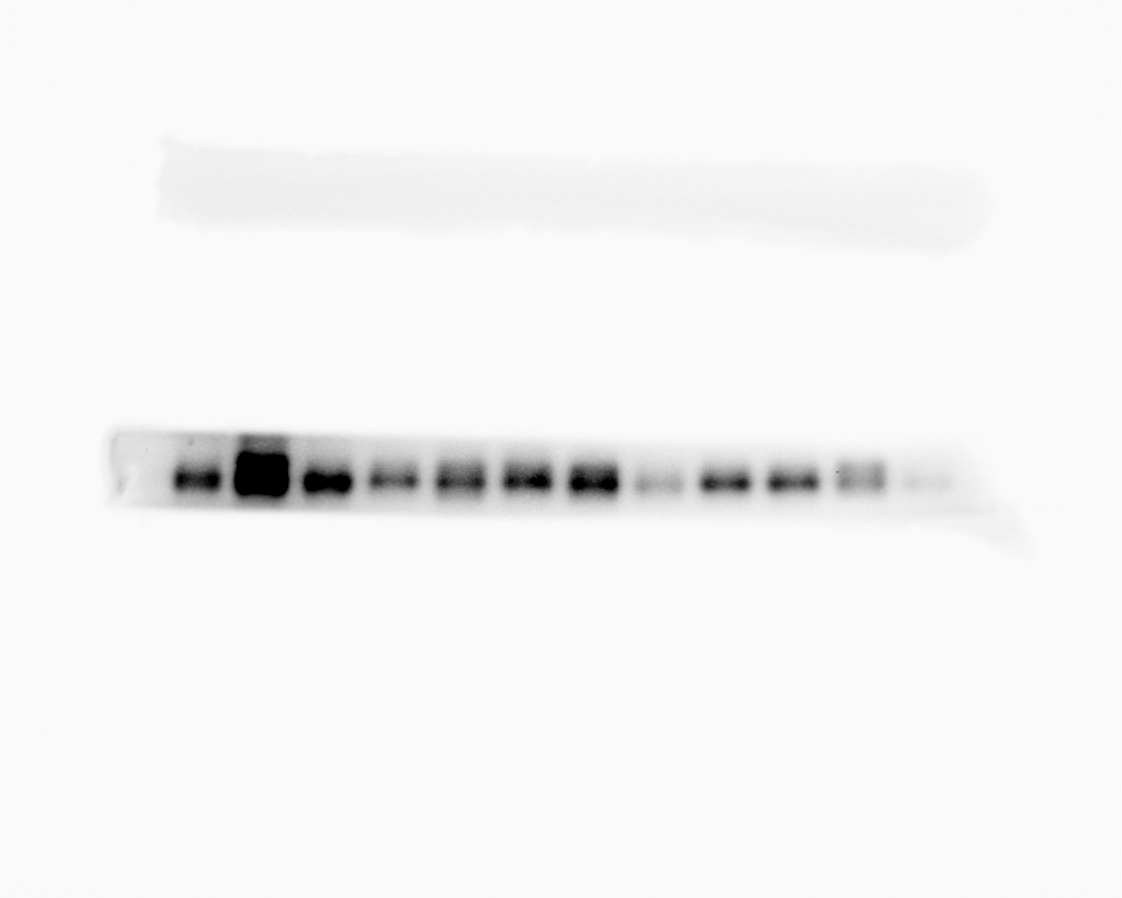

Supplement: Supplemental Information 1 [file peerj-10-13867-s001.zip › uncropped blots in JPEG/figure 6/figure 6C/B6 mice_CQ_24hr_AT_p-GSK3b_2_2018-07-05.jpg]

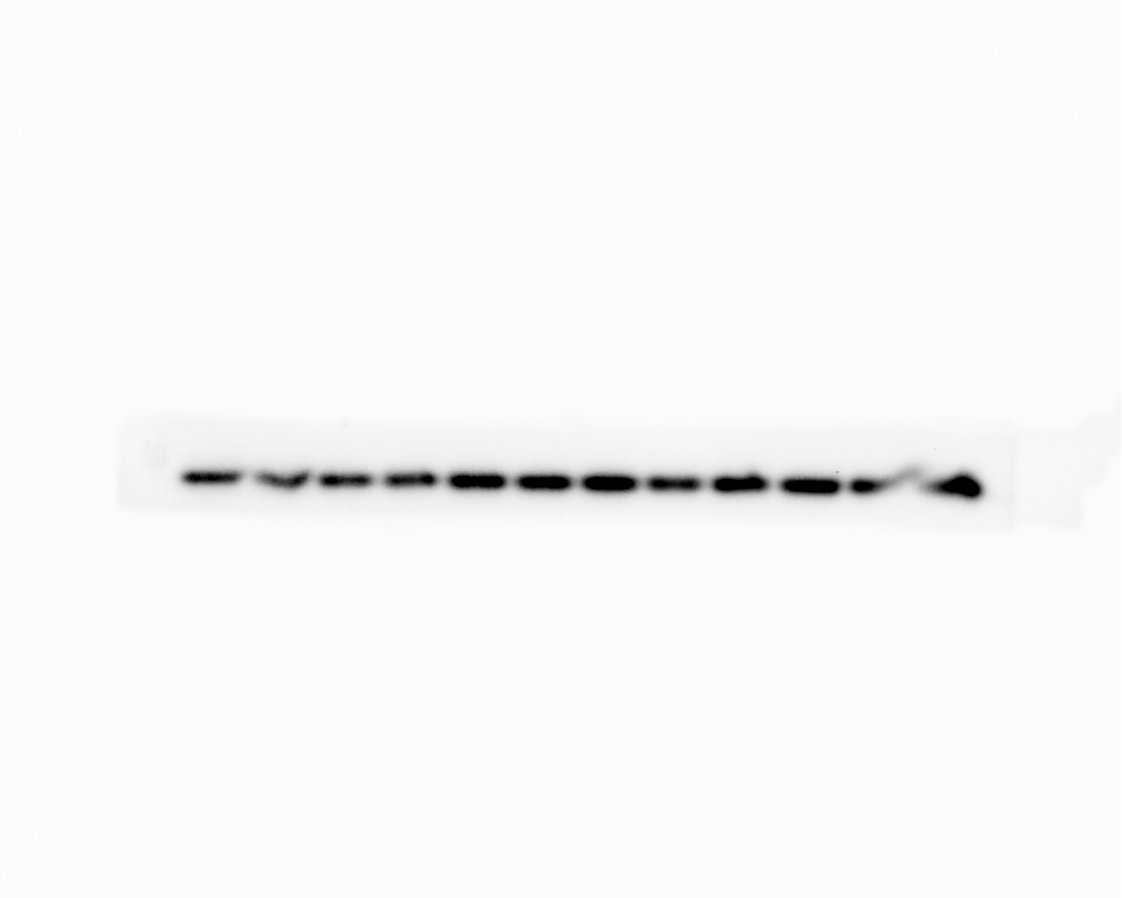

Supplement: Supplemental Information 1 [file peerj-10-13867-s001.zip › uncropped blots in JPEG/figure 7/B6 mice_CQ_24hr_AT_GAPDH for PTEN _ PHLPP1_2018-08-17.jpg]

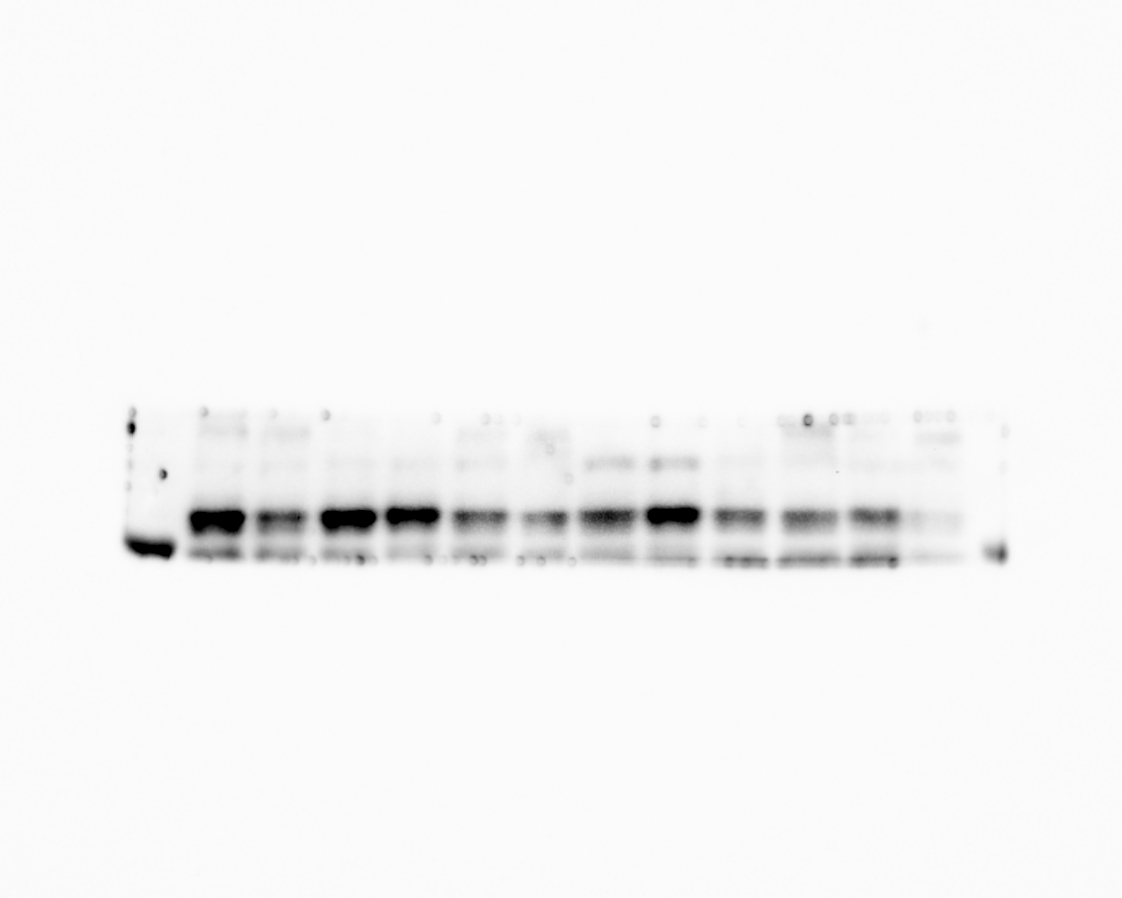

Supplement: Supplemental Information 1 [file peerj-10-13867-s001.zip › uncropped blots in JPEG/figure 7/B6 mice_CQ_24hr_AT_PHLPP1_2018-08-17.jpg]

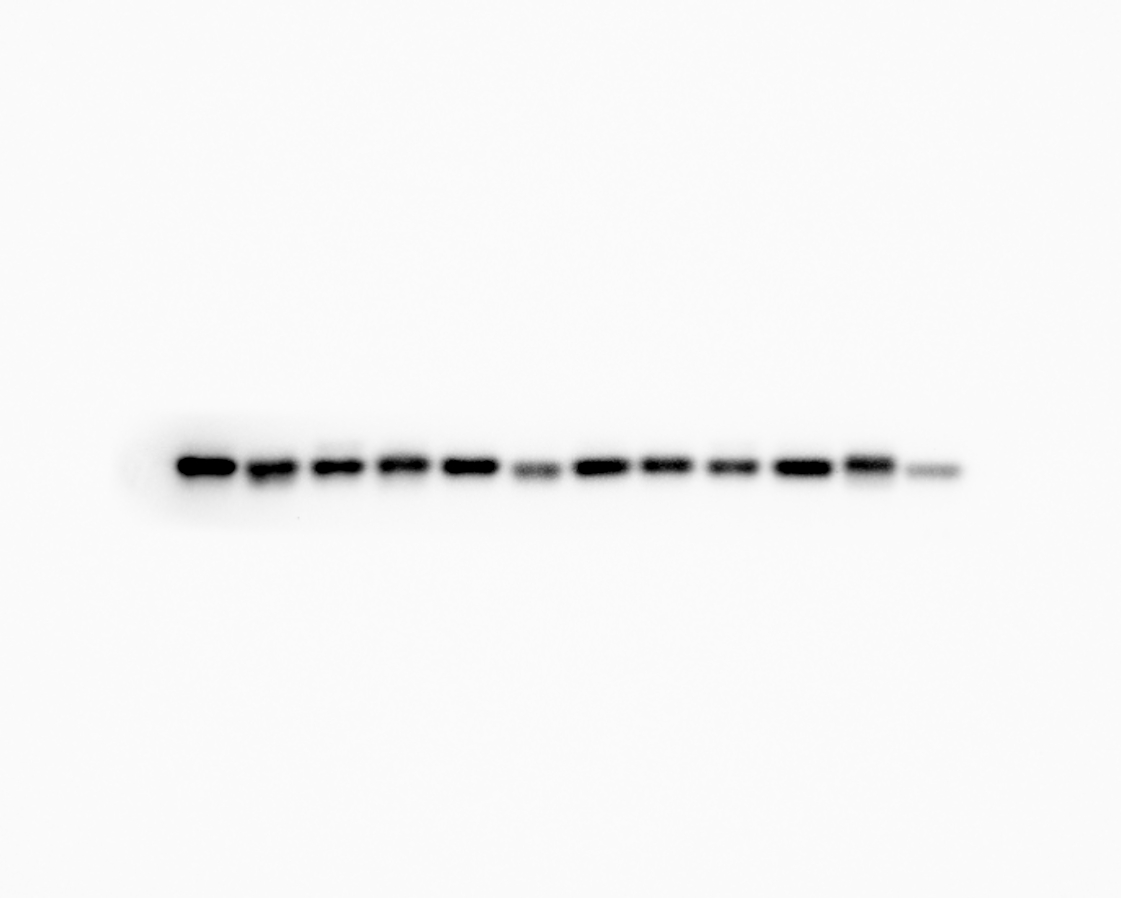

Supplement: Supplemental Information 1 [file peerj-10-13867-s001.zip › uncropped blots in JPEG/figure 7/B6 mice_CQ_24hr_AT_PTEN_2018-08-17.jpg]
